# Polysaccharide- and β-Cyclodextrin-Based Chiral Selectors for Enantiomer Resolution: Recent Developments and Applications

**DOI:** 10.3390/molecules26144322

**Published:** 2021-07-16

**Authors:** Cuong Viet Bui, Thomas Rosenau, Hubert Hettegger

**Affiliations:** 1 Department of Chemistry, Institute of Chemistry of Renewable Resources, University of Natural Resources and Life Sciences, Konrad-Lorenz-Straße 24, Tulln, A-3430 Vienna, Austria; cuongvietbui@students.boku.ac.at (C.V.B.); thomas.rosenau@boku.ac.at (T.R.); 2 Department of Food Technology, Faculty of Chemical Engineering, University of Science and Technology—The University of Danang, Danang City 550000, Vietnam; 3 Johan Gadolin Process Chemistry Centre, Åbo Akademi University, Porthansgatan 3, FI-20500 Åbo, Finland

**Keywords:** amylose, cellulose, chiral selector, chiral stationary phase, chromatography, cyclodextrin, enantiomer separation, polysaccharide

## Abstract

Polysaccharides, oligosaccharides, and their derivatives, particularly of amylose, cellulose, chitosan, and β-cyclodextrin, are well-known chiral selectors (CSs) of chiral stationary phases (CSPs) in chromatography, because they can separate a wide range of enantiomers. Typically, such CSPs are prepared by physically coating, or chemically immobilizing the polysaccharide and β-cyclodextrin derivatives onto inert silica gel carriers as chromatographic support. Over the past few years, new chiral selectors have been introduced, and progressive methods to prepare CSPs have been exploited. Also, chiral recognition mechanisms, which play a crucial role in the investigation of chiral separations, have been better elucidated. Further insights into the broad functional performance of commercially available chiral column materials and/or the respective newly developed chiral phase materials on enantiomeric separation (ES) have been gained. This review summarizes the recent developments in CSs, CSP preparation, chiral recognition mechanisms, and enantiomeric separation methods, based on polysaccharides and β-cyclodextrins as CSs, with a focus on the years 2019–2020 of this rapidly developing field.

## 1. Introduction

**Figure 1:**
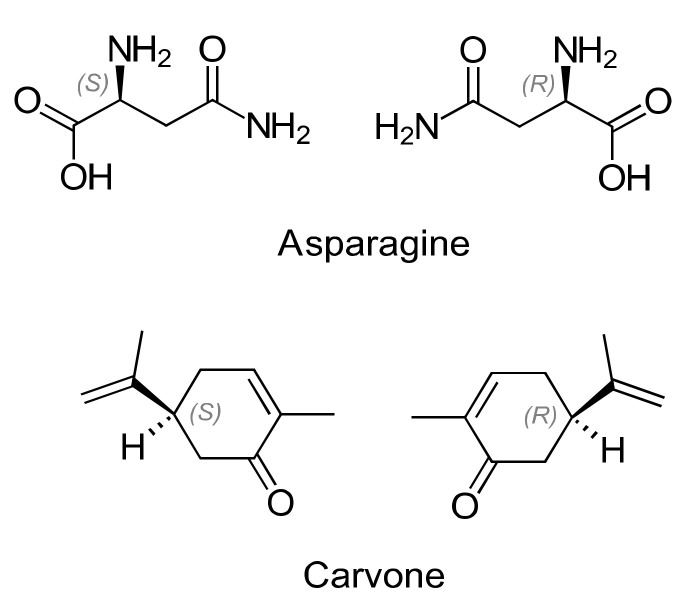
Chemical structures of asparagine and carvone enantiomers.

The equality in the chemical composition and physical–chemical properties are unique features of a pair of enantiomers. However, in a chiral environment, they interact differently with other molecules, such as chiral selectors (CSs), due to the different spatial orientation of the substituent groups around one or more asymmetric centers (*cf.* the well-accepted three-point interaction model [1,2]). The different effects of enantiomers and their different behaviors in biochemical processes are even observable on a macroscopic scale [3,4]. For example, (*R*)-asparagine (d-Asn) gives a sweet taste, while (*S)*-asparagine (l-Asn) exhibits a bitter taste for humans. The same is true for the enantiomeric pair of carvone, with mint odor belonging to (*R*)-carvone and (*S*)-carvone having a caraway smell (Figure 1). In 1848, Louis Pasteur, the pioneer of chirality, introduced the concept of stereoisomerism when he observed the difference in the optical activity of mechanically separated tartrate enantiomers [4].

The importance and need for enantiomer separations in food, drugs, pesticides, and insecticides, continues unabated. The analysis of enantiomers present in food products has attracted great attention, concerning authenticity, legality, and safety. For instance, only l-amino acids must be present in native products of fruit juices, while the presence of their counterparts (d-amino acids) can indicate either an adulteration (external addition) or other problems, which could have occurred during poor preservation [3]. In pharmaceutical applications, a great variety of chiral drugs are administered to humans; it could be that just one out of the two enantiomers shows the desired activities (the so-called *eutomer*), while the other enantiomer is less active (*distomer*), or even induces unwanted or dangerous side effects. This phenomenon is caused by the differences in stereoselective interactions with the chiral biological matrices of living organisms during biochemical processes [4]. Two enantiomeric compounds can exhibit different pharmacokinetics and pharmacodynamics. The control of the enantiomeric purity of drugs thus is a crucial issue in clinical and analytical research, and for regulatory purposes [5].

The global population has been continuously growing, which results in a growing demand for pesticides and insecticides in industrial agriculture. However, chiral pesticides and insecticides also exhibit stereoselective behavior in terms of activity, toxicity, degradation, bioaccumulation, and/or metabolic action. Therefore, by undesired effects, chiral agrochemicals may also be harmful to non-targeted species, including humans, further pollute the environment, and spoil food [6,7].

Stereoselective synthesis for the production of preferably just one single enantiomer has also gained great attention, with efforts towards increasing the yield, improving the enantiomeric/diastereomeric excess (*ee, de*), and simplifying the synthesis processes [4]. Therefore, enantiomeric separation (ES), both in reliable analytical methods and/or on a preparative scale, has attracted interest in the following different fields: pharmaceutical, biomedical, agrochemical, and food sciences, to name only the most prominent examples.

Due to their high stereoselectivity, polysaccharides and β-cyclodextrin derivatives are the main chiral selectors (CSs) for enantiomeric separation [3]. The most prominent analytical application of commercial polysaccharide derivatives, mostly esters and phenyl carbamates, on both analytical and preparative scales, is in (high-performance) liquid chromatography (HPLC) [8]. In addition, these CSs are also employed in supercritical fluid chromatography (SFC), capillary chromatography (CC), and capillary electrochromatography [3,8]. To achieve the maximum performance at separation of the target chiral (enantiomeric) compounds, the chiral recognition mechanisms of polysaccharide and β-cyclodextrin derivatives as CSs still require further investigations [9,10]. The main strategies in further developments of chiral column materials are to introduce new CSs, apply new chromatographic support materials (e.g*.,* core–shell materials), and develop new synthesis approaches for the preparation of chiral stationary phases (CSPs) [3,11]. Also, further improvement in the chromatographic ES performance, enlargement of the versatility, and an extension of the range of applications, need to be addressed [11].

New developments in polysaccharide and β-cyclodextrin derivative-based CSs and CSPs within the last two years have been included in this review article, thus closing the gap between the previous summaries of the field and the current state-of-the-art [12,13,14]. The developments of ES, based on polysaccharide and β-cyclodextrin derivatives as CSs in major application sectors (food, drugs, pesticides, and insecticides), different separation techniques, such as HPLC, SFC, CC, and CEC, as well as recent insights into the chiral recognition mechanism, are included and discussed.

## 2. Cellulose-Based CSPs

### 2.1. Chiral Selector Development

**Figure 2:**
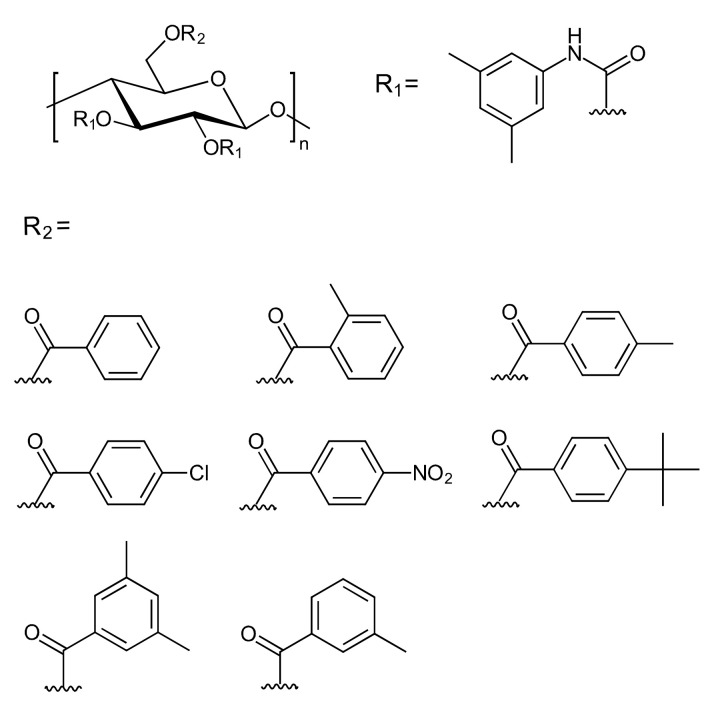
Chemical structure of cellulose 6-*O*-benzoyl-2,3-*O*-(3,5-dimethylphenyl carbamate)-based CSs synthesized by Yin et al. [15].

Celluloses bearing different substituents were frequently shown to possess a higher chiral recognition capacity in comparison to their counterparts bearing the same substituents [15,16,17,18]. Recently, new chiral selectors based on cellulose bearing different substituents were prepared by Yin et al. and Yu et al. [15,19]. A highly regioselective benzoylation of cellulose was used by the former group to prepare cellulose mixed ester derivatives as CSs, according to a two-step route. The primary hydroxy groups of the anhydroglucose (AHG) units in the cellulose backbone (C6) reacted with several different benzoyl chlorides, and then various types of cellulose 6-*O*-benzoyl-2,3-*O*-phenyl carbamates were prepared by the reaction of the 2-OH and 3-OH hydroxy groups of the cellulose 6-*O*-benzoate with 3,5-dimethylphenyl isocyanate. The obtained CSs were then coated onto 3-aminopropyl-modified silica gel as the inert carrier to prepare CSPs. The chemical structures of these CSs are presented in Figure 2.

**Figure 3:**
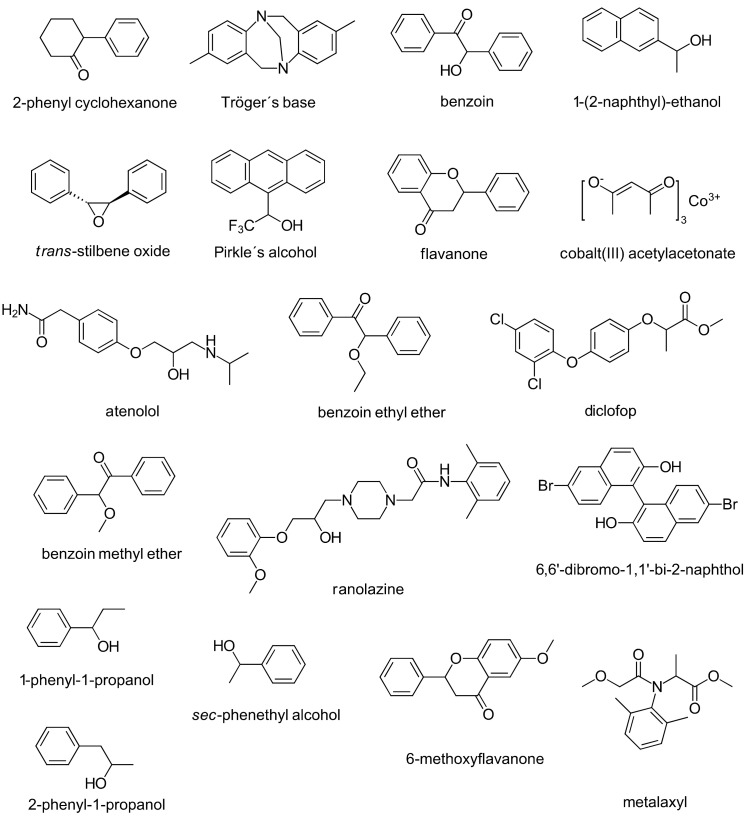
Chemical structures of common chiral analytes used for evaluation of CSPs.

The chiral recognition ability of the developed CSPs was evaluated by employing nine racemates (2-phenyl cyclohexanone, Tröger’s base, benzoin, 1-(2-naphthyl)-ethanol, *trans*-stilbene oxide, Pirkle’s alcohol, cobalt (III) acetylacetonate, atenolol, flavanone) as the respective analytes (Figure 3). Most of the cellulose mixed ester carbamates were shown to have high chiral recognition capability. The CSPs exhibited a higher ES performance than the commercially available Chiralcel^®^ OD column (250 × 4.6, i.d., 10 μm silica gel), due to an electron-donating substituent on the phenyl ring of the ester moiety, such as a 4-*tert*-butyl moiety. Moreover, this research also indicated that the synergy of the hydrophobic helical conformation, weak hydrogen bond donating ability, and appropriate distribution of the substituents of the cellulose derivatives is essential to fabricate high-performance CSPs. 

Cellulose derivatives with organic and alkoxysilane substituents as CSs were synthesized by Yu et al. [19]. Cellulose was first allowed to react with 3,5-dichlorophenyl isocyanate (80 mol% relative to the hydroxy groups of the AHG units) to synthesize cellulose 3,5-dichlorophenyl carbamate, which subsequently reacted with 3-(triethoxysilyl)propyl isocyanate (3.6 mol%). Finally, all the remaining free hydroxy groups were quantitatively derivatized with an excess amount of 3,5-dichlorophenyl isocyanate (120 mol% relative to the residual hydroxy groups). A sol-gel reaction between cellulose 3,5-dichlorophenyl carbamate, carrying small amounts of triethoxypropyl silane groups, and additional tetraethyl orthosilicate was performed, to generate cellulose 3,5-dichlorophenyl carbamate silica hybrids. These hybrid materials were subjected to an end-capping process, with trimethylsilyl chloride as the reagent of choice, to cover all residual hydroxy groups. The chemical structure of this cellulose 3,5-dichlorophenyl carbamate–alkoxysilane hybrid CSP is presented in Supplementary Materials Figure S1. The developed CSP had preferable properties regarding mechanical stability and density. Also, ES evaluation revealed that the hybrid CSP exhibited chiral recognition abilities that were similar to the commercially available Chiralpak^®^ IC (250 × 4.6 mm, i.d., 5 µM silica gel) for ES of the following seven racemates: *trans*-stilbene oxide, 2-phenylcyclohexanone, flavanone, benzoin, Pirkle’s alcohol, cobalt (III) tris(acetylacetonate), and Tröger’s base (see Figure 3).

Anchoring another polymeric CS onto the cellulose backbone, to enhance chiral recognition ability, was investigated by Qian et al. and Gao et al. [20,21]. A new generation of cellulose-grafted copolymers as CS has been discovered by Qian et al. [20]. Polymerization of a right-handed chiral phenyl isocyanate monomer derivative, using an alkyne-terminated palladium (II) complex as an initiator/catalyst, was performed to produce alkyne-terminated right-handed helical poly(phenyl isocyanates), which were then grafted onto 6-deoxy-6-azidocellulose of various chain lengths using copper (I)-catalyzed alkyne–azide cycloaddition (“*click chemistry*”). An increase in the CD intensity value (Δε365), by 111.2%, was found for the right-handed helical poly(phenyl isocyanates) after grafting onto cellulose, indicating a more regular helical conformation. Dansyl chloride-modified d,l-phenylalanine as a fluorescent probe was employed to compare the ES capacity of alkyne-terminated right-handed helical poly(phenyl isocyanates) and their celluloses-grafted counterparts, with the latter exhibiting higher chiral recognition than the former. However, further studies regarding the ES applications of the new cellulosic materials, with a broader set of representative analytes, need to be carried out to confirm this as a general trend.

The chirality of cellulose and β-cyclodextrin may be complementary or synergistic, thus a combination of those two types of materials to enhance the overall ES performance was investigated by Gao et al. [21]. Dialdehyde cellulose derivatives were prepared by a selective oxidation reaction of microcrystalline cellulose with sodium periodate, and then the materials were immobilized onto the surface of amino-modified silica gel by a Schiff’s base reaction and allowed to react with 6-monodeoxy-6-monoamino-β-cyclodextrin to generate silica-bound *bis*-selectors. In the last step of the sequence, all the remaining hydroxy groups of both cellulose and β-cyclodextrin were derivatized with 3,5-dimethylphenyl isocyanate. The product showed good chiral—but also achiral—chromatographic separation performance under normal-phase and reversed-phase modes. The chiral resolution performance was shown to be superior with the silica-bound *bis*-selectors in comparison to the respective single selectors, particularly dialdehyde cellulose (3,5-dimethylphenyl carbamate) or 6-monodeoxy-6-monoamino-β-cyclodextrin *tris*-(3,5-dimethylphenyl carbamate). The performance was evaluated based on eight racemates (flavanone, benzoin ethyl ether, benzoin methyl ether, 6-methoxyflavanon, benzoin, 6,6’-dibromo-1,1’-bi-2-naphthol, metalaxyl, and diclofop, see Figure 3). In terms of achiral separation performance, baseline separation of the aromatic analytes (polycyclic aromatic hydrocarbons, anilines, phenols, phenylates, and aromatic acids) was achieved, based on selective hydrophobic and *π*–*π* electron–donor acceptor interactions.

### 2.2. Material Preparation Developments

Immobilization and coating of cellulose derivatives onto chromatographic supports are the most common approaches to prepare CSPs. A Schiff’s base reaction, as a simple fixation step with a short reaction time, was employed by Gao et al. [22], to prepare cellulose-type CSPs. Microcrystalline cellulose was oxidized with sodium periodate under selective C-2/C-3 bond cleavage to “dialdehyde cellulose” [23], which was then immobilized onto the surface of 3-aminopropyl-modified silica gel by the mentioned Schiff’s base reaction. Subsequently, the remaining free hydroxy groups were fully derivatized with 3,5-dimethylphenyl isocyanate, to generate dialdehyde cellulose 3,5-dimethylphenyl carbamate derivatives that were bound to silica gel as CSP (Supplementary Materials Figure S2). The chromatographic performance of this column material was evaluated by HPLC. The column was highly stable towards organic solvents, which was proven by flushing it with 100% tetrahydrofuran and chloroform for 10 h. The resolution value for 1-(1-naphthyl)ethanol almost remained the same before and after flushing. The chiral column, interestingly, had both chiral and achiral separation abilities. In terms of chiral separation, Pirkle’s alcohol and mandelonitrile were separated on the column in normal-phase mode, while the reversed-phase mode was selected for the successful ESs of ranolazine, benzoin ethyl ether, metalaxyl, and diclofop (see Figure 3). The authors suggested that 20% of acetonitrile in the mobile phase should be used to gain the maximum resolution. The chiral separation ability of dialdehyde cellulose 3,5-dimethylphenyl carbamate was slightly lower than the one of cellulose *tris*-3,5-dimethylphenyl carbamate. The dependence of ES on the degree of oxidation of the dialdehyde celluloses was not studied. Based on the retention mechanism of achiral aromatic compounds, the *π*–*π* interaction, *π*–*π* electron–donor-acceptor interaction, and hydrogen bonding interaction significantly contribute to achiral recognition and separation. Polycyclic aromatic hydrocarbons were separated within 10 min in normal-phase mode, while aromatic acids in reversed-phase mode were better separated on the developed column than on a standard C_18_ column. The facile preparation of the stationary phase, which had both chiral and achiral separation ability, was seen as a tremendous advantage.

A similar Schiff base reaction was also used by Gao et al. [24] to develop new chiral column materials. After anchoring dialdehyde cellulose onto the surface of 3-aminopropyl-modified silica gel, the remaining aldehyde groups were further oxidized to convert them to carboxylic acid groups, to obtain dicarboxyl cellulose [25] that is covalently bonded to silica gel. In a similar approach, dialdehyde cellulose on 3-aminopropyl-modified silica gel reacted with (*S*)-*α*-phenylethylamine, and was subsequently oxidized to obtain (*S*)-*α*-phenylethylamine dicarboxyl cellulose. The authors indicated that those two modified celluloses could be applied as CSPs in hydrophilic interaction chromatography (HIC) and ion-exchange chromatography (IEC). Due to their excellent hydrophilic chromatographic performance, both dicarboxyl cellulose and (*S*)-*α*-phenylethylamine dicarboxyl cellulose showed similar, or even better, separation capabilities than a commercially available GS-120–5-APS amino column. The method is promising for the preparation of chromatographic stationary phase materials to be used in several ways, under normal-phase, reversed-phase, hydrophilic interaction, and ion-exchange chromatographic modes.

Another type of highly selective reactions in the framework of “*click chemistry*” is the “*thiol-ene* reaction”, as a method to immobilize cellulose derivatives onto silica gel as an inert carrier to prepare CSPs. Yin et al. [26] have synthesized cellulose 3,5-dimethylphenyl carbamates with acrylate groups with different degrees of substitution, in a controlled way. The chiral selectors were subsequently immobilized onto mercaptopropyl-modified silica gel. The chemical structure of the CS and CSP are presented in Supplementary Materials Figure S3. The developed CSPs tolerated strong solvents, such as hot pyridine, chloroform, *N*,*N*-dimethylformamide, dimethyl sulfoxide, etc., due to the quite stable thioether bonds. Therefore, the CSPs could be applied to ES, with a wide range of mobile phases, including “standard” solvents, non-standard solvents, and reversed-phase solvents. Concerning the chiral recognition ability, the immobilized CSP with an acrylate DS of 0.012 (which corresponds to one acrylate group per 83 AHG units) was shown to have a similar, or better, chromatographic performance than the respective coated CSP. The set of analytes in this study comprised the following eight racemates: 2-phenylcyclohexanone, Tröger’s base, d,l-*sec*-phenethyl alcohol, flavanone, 1-(2-naphthyl)-ethanol, benzoin, cobalt (III) acetylacetonate, and 2-phenyl-1-propanol (see Figure 3). Notably, the flavanone racemates had a higher resolution on the immobilized CSP than on the coated CSP.

Another chiral column material with 3,5-dichlorophenylcarbamoylated cellulose immobilized onto silica gel was developed by Li et al. [27], and applied to the ES of the following six pesticides: triticonazole, hexaconazole, tebuconazole, triadimefon, metalaxyl, and benalaxyl (Supplementary Materials Figure S4). A stepwise method was used to anchor cellulose derivatives onto silica gel, applying “*thiol-ene*” chemistry. Allyl isocyanate was employed as a bifunctional coupling agent; the isocyanate was first allowed to react with the hydroxy groups of the AHG units on the cellulose backbone, and the alkene subsequently reacted with 3-mercaptopropyl-modified silica gel in a radical-mediated “*thiol-ene addition*” reaction, which immobilized the cellulose 3,5-dichlorophenyl carbamate on the surface of the silica gel. A method for fungicide analysis, by a combination of liquid chromatography and MS towards selective qualification and sensitive quantification of the chiral analytes, was also developed, which used 0.1% formic acid in acetonitrile as the mobile phase.

An organic polymer as the inert chromatographic support was developed by Echevarría et al. [28], prepared in situ in pretreated fused silica capillaries, by thermally induced polymerization of 2-hydroxyethyl methacrylate and ethylene glycol dimethacrylate in the presence of the binary porogenic mixture of 1-dodecanol and cyclohexanol, which produced a network and pore size that was adequate for proper coating of the bulky CS, as well as for obtaining good mobile-phase permeability. The use of the polar monomer 2-hydroxyethyl methacrylate allowed homogeneous adsorption of the polar CS onto the monolithic surface. Cellulose *tris*-(3,5-dimethylphenyl carbamate) was then coated onto the synthesized chromatographic support, to obtain a coated-type monolithic chiral column material. The ES was tested with nine chiral acidic, neutral, and basic compounds (profens, β-blockers, pesticides, and others) under normal-phase conditions, with different ratios of *n-*hexane and 2-propanol. The developed CSPs provided good ES, since all of the analytes were at least partially, or even completely, resolved with enantioselectivity values (*α*) up to 7.1 and enantiomeric resolutions (*R_s_*) up to 2.4 at short retention times. The addition of acidic or basic auxiliaries was necessary to improve the enantiomeric resolution of most of the racemates.

### 2.3. Chiral Recognition Mechanism Studies

Elucidation of the chiral recognition mechanism is a very intriguing aspect that can give much support to the preparation and/or selection of an adaptive CSP for given targets. Carradori et al. [29] have introduced two homologous series of chiral sulfoxides (Supplementary Materials Figure S5), with either an amide or a methyl ester group *ortho* to the sulfoxide, and alkyl moiety bound to the sulfur atom, based on the same 2-(sulfinyl)benzoyl core as the prototype of new selectants. The enantioselectivity was tuned by the type of unbranched alkyl chain, varying in length from one to five carbon atoms, to better understand the ES mechanism of the cellulose *tris*-(3,5-dichlorophenyl carbamate) as CS in HPLC.

According to these experiments, the 2-(sulfinyl) benzoyl scaffold was an ideal model for studying the chiral recognition mechanism. The highest ES was achieved for the amide derivative with an *n*-pentyl chain. The insertion of different alkyl groups at the sulfinyl moiety, along with the insertion of an amide group, gave significant differences in enantioselectivity, but no change in the enantiomeric elution sequence; the (*R*)-enantiomer always eluted before the (*S*)-enantiomer. Interestingly, the chiral discrimination was reverted by the insertion of a methyl ester group, resulting in a change in the enantiomer elution sequence. It was interesting to observe that the operative column temperature also induced the reversal of the order of elution.

### 2.4. Applications of Commercial Cellulose-Based Chiral Columns

The composition of the mobile phase is the most significant factor in method development, concerning the ES of a certain stationary phase in HPLC. Varying the mobile phase is also useful in analyzing the interactions between the analytes and CSs, to better understand the underlying chiral recognition mechanism. 

Regarding the food sector, a method for separation of *α*- and *γ*-linolenic acid positional isomers (Supplementary Materials Figure S6)—which was a major challenge in previous methods—by liquid chromatography with Lux^®^ i-Cellulose-5 (250 × 4.6 mm, i.d., 5 μm silica gel, Table 1), has been developed by Ianni et al. [30]. They applied a reversed-mode mobile phase (acetonitrile/10 mM aqueous ammonium acetate, 60:40, *v:v*), pH 6.0, 25 °C, and a 0.5 mL/min flow rate as the optimal conditions for the separation of the two isomers, with the highest selectivity and resolution being *α* = 1.10 and *R_S_* = 1.21, respectively. The developed method was successfully transferred to an LC-MS platform.

From the viewpoint of drug analysis, a new method for the ES of eight imidazole-type antifungal drugs (miconazole, econazole, isoconazole, sulconazole, butoconazole, fenticonazole, sertaconazole, and ketoconazole, see Supplementary Materials Figure S7), on commercially available Chiralpak^®^ IC columns (250 × 4.6 mm, i.d., 5 µM silica gel, Table 1), has been reported by Zhang et al. [31]. The impact of three different mobile-phase modes (normal, polar organic, and reversed) was investigated. The Chiralpak^®^ IC column well separated almost all the analytes. Basic additives to the polar organic phase played a crucial role in the ES of imidazole antifungal drugs. Also, the effect of buffer salt in the aqueous phase of the reversed-phase mode was important. In normal-phase mode, a successful ES was obtained for seven analytes (except for ketoconazole) within a comparably shorter retention time. Although the ketoconazole enantiomers were not resolved in the normal-phase mode, the baseline ES of the analytes was achieved within 30 min under the reversed-phase mode.

Another commercially available chiral cellulose-based column, Chiralpak^®^ IB (250 × 4.6 mm, i.d., 5 μm silica gel, Table 1), was employed by Li et al. for the ES of six β-adrenergic blockers (atenolol, bevantolol, carteolol, esmolol, metoprolol, and propranolol, Supplementary Materials Figure S8), using a mixture of *n*-hexane with either ethanol or isopropanol/0.1% diethylamine as a normal-phase eluant [32]. Ethanol allowed for better resolution of the six analytes than isopropanol. All the investigated analytes were baseline-separated when the proportion of ethanol in the mobile phase was in the range of 10–15%. Molecular interaction studies indicated that hydrogen bonds, *π*–*π* interactions, hydrophobic interactions, and steric effects contribute to the ES of those compounds.

Polysaccharide derivatives as CSPs efficiently supported the synthesis and isolation of new chemicals in terms of ES, thus shortening the timelines of chiral drug discovery and development, as suggested by Cerra et al. [33]. A commercial chiral column, Chiralpak^®^ IB (250 × 4.6 mm, i.d., 5 µM silica gel, Table 1), was used to assist the isolation of chiral imidazolines (Supplementary Materials Figure S9) by HPLC.

In this study, nine out of ten of the investigated enantiomeric pairs were successfully separated under reversed mobile-phase conditions (eluant: 50% water and different contents of acetonitrile and methanol as modifiers, pH 7.5 with 40 mM ammonium acetate), with *α* < 1.10 and *R_s_* up to 2.31. The authors also pointed out that the aqueous mobile phases were employed to further comply with the paradigms of green chemistry. The *π*-*π* stacking interactions between CS and the analytes significantly contributed to the retention and chiral recognition. Interestingly, an *n*-hexane/chloroform/ethanol mixture (88:10:2, *v:v:v*) as a non-standard mobile phase allowed for almost baseline ES (*α* = 1.06; *R_s_* = 1.26) of the target analytes, which were not resolved under reversed mobile-phase conditions.

Besides amylose-based materials, the commercially available and well-established cellulose-type chiral columns are frequently used in method development for the ES of chiral chemicals. Also, their respective chromatographic performance has been compared. Clear general statements and conclusions are sometimes difficult to derive, as the separation performance strongly depends on the respective analyte/selector pair and the mobile-phase composition, as well as other chromatographic parameters, such as the silica dimensions or temperature.

A challenge for the separation and quantification of the synthetic racemic drug medetomidine (Supplementary Materials Figure S10), used as a doping agent, from complex biological matrices, has been recently resolved by Karakka Kal et al. [34]. The ES behavior on different commercial chiral columns was studied in detail, comparing Lux^®^ Amylose-1, Lux^®^ Amylose-2 (Table 2), Lux^®^ Cellulose-1, Lux^®^ Cellulose-2, Lux^®^ i-Cellulose-5, Chiralcel^®^ OD-RH, and Chiralcel^®^ OJ-3R (Table 1); the parameters were 150 × 4.6 mm, i.d., 5 μm silica gel for all the columns. The Chiralcel^®^ OJ-3R column was selected and employed for routine analysis instead of protein-based chiral columns, which had been commonly used in previous methods.

Ammonium hydrogen carbonate as buffer salt was preferable for successful ES. Baseline ES was obtained with a mobile phase composed of 10 mM aqueous ammonium bicarbonate solution and acetonitrile, at a 70:30 (*v:v*) ratio. The developed method is applicable for the selective determination and sensitive quantification towards medetomidine enantiomers, while the linearity of quantitation was obtained over the range of 0–20 ng/mL equine plasma. The limit of quantification for both enantiomers was 0.2 ng/mL. This method was considered as the standard for the identification and quantification of medetomidine enantiomers in biological matrices that are used in drug testing and analysis.

The analytical chromatographic development of normal-phase HPLC methods (*n*-hexane/alcohol/diethylamine and *n*-heptane/alcohol/diethylamine), in parallel with a comparative study on the ES of a broad set of commercially available chiral column materials, with isopulegol-based β-amino lactones and β-amino amides as the given targets, was carried out by Tanács et al. [35]. The respective columns used were Chiralpak^®^ IA, Chiralpak^®^ IB, Chiralpak^®^ IC, Chiralpak^®^ ID, Chiralpak^®^ IE, Chiralpak^®^ IF, Chiralpak^®^ IG, Chiralpak^®^ AD-H, and Chiralpak^®^ OD-H (all 250 × 4.6 mm, i.d., 5 µM silica gel for all the columns, Table 1). The chemical structures of the CSPs, the position of the substituents on the phenyl carbamate moiety, the types of CSPs in terms of coated and immobilized CSs, and the chromatographic conditions, were studied in detail, concerning their effects on the ES. Regarding the structure of CSPs, the nature of the selector backbone (cellulose and amylose), together with the nature and position of the substituents of the phenyl carbamate moiety, had a dramatic influence on both the ES performance and the elution order. Due to the *π*-*π* acceptor-type character, *tris*-(3,5-dichlorophenyl carbamate) substituents on the cellulose and amylose backbone resulted in stronger retention than *tris*-(3,5-dimethylphenyl carbamate) substituents. Also, the elution sequence was affected by the structure of the substituents. The chiral recognition of the given analytes was generally better with the amylose *tris*-(3-chloro-5-methylphenyl carbamate) than with the *tris*-(3-chloro-4-methylphenyl carbamate) derivative.

The well-known trend that the immobilized polysaccharide derivatives as CSPs are very robust, but their chromatographic performance is typically somewhat lower than that of their coated counterparts under the same mobile-phase conditions, was also confirmed. However, this drawback can be overcome by the much broader range of solvents that are applicable as mobile phase with immobilized selectors. Regarding the composition of the mobile phase, the use of either 2-propanol or ethanol in the mobile-phase mixture had a significant impact on ES, while the effect of a change from *n*-hexane to *n*-heptane was less pronounced.

A comparison between commercial chiral columns under the trademark Chiralpak^®^ AD-H and Chiralcel^®^ OJ-H (250 × 4.6 mm i.d., 5 µM for all the columns, Table 1) for the ES of the drugs clausenamidone and neoclausenamidone (Supplementary Materials Figure S11), including thermodynamic parameters, was performed by Luo et al. [36]. Optimum separation of the enantiomers was obtained on Chiralcel^®^ OJ-H, with a mixture of *n*-hexane and isopropanol (80:20, *v:v*) as the eluant. The enantioselectivity (*α*) and resolution (*R_s_*) for clausenamidone/neoclausenamidone were 1.90/1.38 and 9.06/7.08, respectively. Thermodynamic studies showed that the ES of clausenamidone and neoclausenamidone was enthalpically controlled with *π*-*π* interactions and hydrogen bonding between CSPs, and the analytes playing an important role in the chiral recognition, which was supported by a docking study.

The comparison between the different types of commercial chiral columns—Chialpak^®^ AD-H (250 × 4.6 mm, i.d., 5 µM silica gel), Chialcel^®^ OD-H (250 × 4.6 mm, i.d., 5 µM silica gel), Chiralcel^®^ OJ (250 × 20 mm, i.d., 10 µM silica gel) and Chialpak^®^ AS (250 × 20 mm, i.d., 10 µM silica gel) (Table 1), and the selection of a suitable analytical method for preparative ES of 6-(4-aminophenyl)-5-methyl-4,5-dihydro-3(2*H*)-pyridazinone (Supplementary Materials Figure S12) was reported by Cheng et al. [37]. Chiralcel^®^ OJ was the material of choice for ES in HPLC, with pure methanol as the eluant. The enantioselectivity and resolution reached *α* = 1.71 and *R_s_* = 5.47, respectively, under optimum conditions. The Chiralpak^®^ AS column was the best option for the ES of the analyte (*α* = 1.81 and *R_s_* = 6.51) in SFC, with scCO_2_/methanol as the mobile phase. An opposite elution order was observed in the two analytical approaches (HPLC with Chiralcel^®^ OJ column and SFC with Chiralpak^®^ AS column). The authors discussed that for large-scale chiral separation, either SFC should be chosen due to its higher productivity, or HPLC because of its simpler mobile phase.

A method for the ES of limonene-based bicyclic 1,3-aminoalcohols and 1,3,5- and 1,3,6-aminodiols was reported by Orosz et al. [38]. Different types of commercial chiral columns were employed, namely, Lux^®^ Cellulose-2, Lux^®^ Cellulose-3, Lux^®^ Cellulose-4, Lux^®^ i-Cellulose-5 (Table 1), Lux^®^ Amylose-1, Lux^®^ Amylose-2 (250 × 4.6 mm i.d., 5 μm silica gel for all the columns) (Table 2) and Lux^®^ Cellulose-1 (250 × 4.6 mm i.d., 3 μm silica gel) (Table 1), under normal-phase HPLC and SFC conditions. The authors optimized the ES, based on the composition of the mobile phase, the operating temperature, the interaction between the analytes and CSs, and the structure of the analytes and polysaccharide derivatives, concerning retention, enantioselectivity, resolution, and elution sequence as the given parameters. Studies on the effects of the mobile-phase composition had revealed that an increase in the apolar component in the mobile phase (*n*-hexane or CO_2_) resulted in a pronounced increase in the retention time. The nature of the alcohol modifiers was also taken into consideration, as longer chain and bulky alcohol modifiers generally resulted in an increase in retention, with only a minor effect on enantiomeric discrimination. Both enantioselectivity (*α*) and resolution (*R_s_*) were shown to be most affected by the use of 2-propanol and methanol under normal-phase HPLC and SFC conditions, respectively. Studies on the interactions of the analytes with the CSs indicated that hydrogen bonding and *π*-*π* interaction significantly contributed to the chiral discrimination.

An HPLC-based analytical method for the determination of the chiral and achiral impurities of ivabradine (Supplementary Materials Figure S13)—a heart rate-lowering drug—on polysaccharide-type CSPs, namely, Lux^®^ Amylose-1, Lux^®^ Amylose-2, Lux^®^ i-Amylose-1 (Table 2), Lux^®^ Cellulose-1, Lux^®^ Cellulose-2, Lux^®^ Cellulose-3, and Lux^®^ Cellulose-4, (150 × 4.6 mm, i.d., 5 μm silica gel for all the columns) (Table 1), was presented by Ferencz et al. [39]. Mobile phases, consisting of 0.1% diethylamine in methanol, 2-propanol, and acetonitrile, were employed to screen the ES characteristics of seven chiral columns. Cellulose *tris*-(3-chloro-4-methylphenyl carbamate) proved to be the most suitable CSP for a mobile phase of methanol, in which all the impurities eluted before *S*-ivabradine. All the ivabradine compounds were baseline-separated under the following chromatographic conditions: 0.06% (*v:v*) diethylamine in methanol/acetonitrile (98:2, *v:v*), 0.45 mL/min flow rate and 12 °C operative temperature. The developed method was shown to be reliable, linear, precise, and accurate for the determination of impurity concentrations as low as 0.05% in *S*-ivabradine samples, after validation according to the International Council for Harmonisation Q2 (R1) guidelines, and successful application with commercial tablet samples.

Method development towards the reduction in hazardous mobile-phase components, in agreement with green chemistry principles, was taken into consideration by Tantawy et al. [40]. The authors employed different commercial chiral columns with smaller dimensions (50 × 4.6 mm, i.d., 5 μm silica gel), namely, Lux^®^ Cellulose-1, Lux^®^ Cellulose-2, Lux^®^ Cellulose-3, Lux^®^ Cellulose-4, see Table 1), and Lux^®^ Amylose-2 (Table 2), for the ES of guaifenesin enantiomers (Supplementary Materials Figure S14) in ambroxol hydrochloride binary mixtures. The targets of high enantioselectivity and low volume of mobile phase were achieved with coated cellulose *tris*-(3,5-dimethylphenyl carbamate) as CSP. A binary mixture of ethanol and water as a mobile phase, and linear gradient elution from 20 to 70% ethanol within 6 min were the optimum chromatographic conditions. In comparison with previous methods, the authors stated that the solvent waste was reduced by about 60%, and only 1 mL of ethanol was needed in the mobile phase.

Cutillas et al. [41] quantified the chiral pesticides cyhalothrin and metalaxyl by SFC hyphenated to MS detection. The ES of those compounds in SFC was performed on the commercial chiral columns Lux^®^ Cellulose-1 (Table 1) and Lux^®^ Amylose-3 (Table 2) (250 × 4.6 mm, i.d., 5 μm silica gel for all the columns). The developed method was fast, robust, and efficient for the stereoselective separation and quantification of the analytes.

For method development, a comparative study regarding the separation of *N*-acetyl-d,l-cysteine, a chiral thiol, after precolumn derivatization with *o*-phthaldialdehyde and primary amines on different types of commercial chiral columns, namely, Chiralcel^®^ OD-3R, Chiralpak^®^ AS-3R, Chiralpak^®^ AD-3R, Chiralpak^®^ AY-3R, Chiralcel^®^ OJ-3R, Chiralpak^®^ AZ-3R, and Chiralcel^®^ OZ-3R (150 × 4.6 mm, i.d., 3 μm silica gel for all the columns, see Table 1), in reversed-phase mode, was performed by Douša [42]. The best results were obtained with the Chiralcel^®^ OZ-3R column with an *R_s_* > 2.5. Also, the developed method fulfilled the guidelines of the International Council for Harmonisation (ICH) of Technical Requirements for Pharmaceuticals for Human Use, in terms of linearity, the limit of detection, the limit of quantification, precision, accuracy, and selectivity. Therefore, the method was applied to the determination of the optical purity of commercial *N*-acetyl-d,l-cysteine samples.

## 3. Amylose-Based CSPs

### 3.1. Chiral Selector Development

Regioselective synthesis is the most effective method that is employed to produce amylose derivatives bearing different substituents, to improve chiral recognition ability. Dai et al. [43] have applied temporary protection of the primary hydroxy groups of the AHG units in amylose, with trityl chloride as the protection reagent, to prepare seven amylose derivatives, which each have two different substituents, including 3,5-dimethylphenyl, 3,5-dichlorophenyl, 4-chlorophenyl, and cyclohexyl carbamate groups (Supplementary Materials Figure S15). The amylose derivatives were coated onto 3-aminopropyl-modified silica gel, and subsequently packed into empty columns by the slurry method. The chiral recognition ability of the developed CSPs significantly depended on the substitution pattern, in particular the positions of the respective substituents (at C2, C3, or C6). The crucial role of the substituents on the aromatic moieties, in terms of their nature, position, and number, was shown. The carbamoylated amylose derivatives bearing electron-withdrawing *para*-chloro-substituents at the phenyl carbamate at C2 and C3, possessed comparably higher chiral recognition performances than the others. The developed chiral column materials provided better ES for some racemates in comparison to the commercial chiral column Chiralpak^®^ AD (250 × 4.6 mm, i.d., 10 µM silica gel).

**Figure 4:**
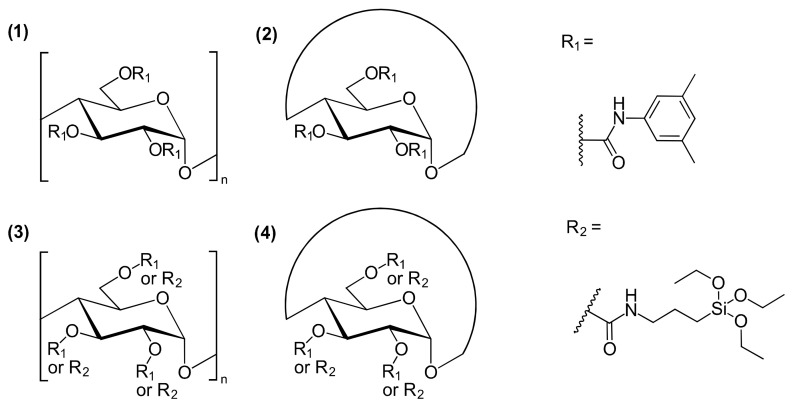
Chemical structures of linear amylose 3,5-dimethylphenyl carbamate for coating (**1**), cyclic amylose 3,5-dimethylphenyl carbamate for coating (**2**), linear amylose 3,5-dimethylphenyl carbamate for immobilization (**3**), and cyclic amylose 3,5-dimethylphenyl carbamate for immobilization (**4**), compared in the study of Ryoki et al. [44].

Local chain conformation of polysaccharide derivatives plays an important role in chiral recognition, which was also shown in the research of Ryoki et al. [44]. The authors compared cyclic amylose 3,5-dimethylphenyl carbamates and linear amylose 3,5-dimethylphenyl carbamates for ES, as both coated and immobilized CSPs (Figure 4).

The local helical structure of amylose plays an important role concerning chiral separation ability, while coated cyclic linear amylose derivatives were shown to have significant differences in ES ability. However, it was noteworthy that the chiral separation ability of both the immobilized cyclic and linear amylose derivatives was quite similar if the number of linkage points generated in the immobilization process was high.

### 3.2. Material Preparation Developments

The immobilization and coating of CSs onto chromatographic supports are the two general approaches to prepare CSPs. Their advantages and drawbacks are nowadays well established and understood. The immobilized CSPs are suitable for a wide range of eluents, while their chiral recognition ability is usually lower than that of their coated counterparts, due to reduced flexibility. Maisuradze et al. [45] have provided a deeper understanding of the differences between immobilized and coated amylose *tris*-(3-chloro-5-methylphenyl carbamate), in terms of the effects of mobile-phase composition and operative temperature on the retention behavior of enantiomers, their separation factor, and enantiomer elution order for chiral organic acids. They used thermodynamic parameters, such as Gibb’s free energy, the standard molar entropy, and the standard molar enthalpy of analyte transfer from the mobile phase to the CSP, to explain the differences observed in the ES ability between the two types of CSPs.

The effect of the coating amount on chiral recognition was the topic of research by Wang et al. [46]. An increase in the coating amount of amylose *tris*-(3,5-dimethylphenyl carbamate) resulted in a higher chiral resolution of (±)-Corey lactone diol (Supplementary Materials Figure S16), under both HPLC and SFC conditions. The increase in productivity, regarding the high sample loading capacity, high ES efficiency, and reduced solvent consumption of the latter technique, were considered as well.

Wang et al. suggested that a CSP with 30% coated amylose *tris*-(3,5-dimethylphenyl carbamate) is suitable for semi-preparative scale, under both HPLC and SFC conditions. With such a stationary phase, preparative performances reached up to 1.54 and 1.25 kg racemate/kg CSP/day, applying HPLC and SFC, respectively. A proper eluent for preparative chromatography was also evaluated. Due to the high resolution of enantiomers and the high solubility of (±)-Corey lactone diol, a polar organic mobile phase was chosen for HPLC, while methanol was suggested as the CO_2_ modifier of choice in SFC mode.

A new generation of CSPs was obtained, by coating amylose *tris*-(3-chloro-5-methylphenyl carbamate) onto 5 μm silica gel packed in columns (250 × 4.6 mm, i.d.), as inter alia reported by Dascalu et al. [47]. The enantiomer separation of 1-aryl-5-aryl-pyrrolidin-2-one derivatives and the related commercial drugs (Supplementary Materials Figure S17), was compared to different types of other commercial amylose-type chiral columns, such as Chiralpak^®^ AD, Chiralpak^®^ IA, and Lux^®^ Amylose-2, (each 250 × 4.6 mm, i.d., 5 μm silica gel) under SFC conditions, with either 30% methanol or ethanol as the CO_2_ modifiers. The newly developed column material provided the best resolution of most analytes in comparison to the commercial amylose-type chiral columns.

### 3.3. Chiral Recognition Mechanism Studies

The individual interactions of the analytes and CSPs are the most important factors to study about chiral recognition mechanisms. These interactions are also addressed when the enantiomer discrimination ability between different polysaccharide derivatives as CSs is compared. Upmanis et al. [48] reported an extensive study using the six commercial chiral columns Lux^®^ Amylose-1, Lux^®^ Amylose-2 (Table 2), Lux^®^ Cellulose-1, Lux^®^ Cellulose-2, Lux^®^ Cellulose-3, and Lux^®^ Cellulose-4 (Table 1) (150 × 4.6 mm, i.d., 5 μm silica gel for all the columns), under normal and polar organic mobile-phase conditions in HPLC for the ES of 15 structurally different 4C-substituted pyrrolidin-2-one derivatives (Supplementary Materials Figure S18).

The authors stated that the steric effects and helicity related to the polysaccharide backbone play a crucial role in chiral recognition of the analytes, which was concluded from a better separation on the amylose derivatives than on the cellulose derivative CSPs. For example, amylose *tris*-(5-chloro-2-methylphenyl carbamate) as CS separated 14 out of 15 of the investigated chiral compounds, with *R_s_* > 2.0. A complex chiral environment, induced by the helical structure of the amylose derivatives, led to increased chiral recognition. The structure of the respective racemates also played an important role in chiral recognition; the presence of bulky groups at the nitrogen position in the pyrrolidine-2-one moiety and/or at the 4C position increased their chiral resolution. Both the retention and resolution of the studied analytes depended significantly on the nature of the alcohol solvent in the mobile phase.

### 3.4. Applications of Commercial Chiral Columns Based on Amylose

In the field of food analysis, Zhao et al. [49] developed a method for combined chiral liquid chromatography and tandem MS, to obtain selective enantio-qualification and sensitive enantio-quantification of six chiral pesticides in functional foods (Supplementary Materials Figure S19). The commercial chiral column, Chiralpak^®^ IG (250 × 4.6 mm, i.d., 5 µM silica gel, Table 2), and a reversed mode mobile phase were employed in liquid chromatography, for the separation of these analytes. The developed method showed an excellent analytical performance, which was evaluated and validated according to the European Union SANTE/11945/2015 guidelines, regarding specificity, linearity, matrix effects, stability, sensitivity, and trueness.

The enantiomers of new chiral psychoactive substances may differ in their pharmacological and pharmacokinetic effects, which makes it necessary to separate the two enantiomers to gain a deeper understanding of the individual effects. The ES of a set of 112 psychoactive substances that were obtained by syntheses, and from different sources during the years 2010–2018, was the objective of a study by Kadkhodaei et al. [50], in which the commercial chiral column material Lux i-Amylose-1 (250 × 4.6 mm, i.d., 5 μm silica gel, Table 2) was applied under isocratic HPLC mobile-phase conditions (*n*-hexane/isopropanol/diethylamine, 90:10:0.1, *v:v*:*v*). A total of 79 of the investigated analytes were successfully separated, and 37 of them were baseline-separated. The number of the baseline-separated pairs was improved by 27 when the proportion of *n*-hexane in the eluant was increased (*n*-hexane/isopropanol/diethylamine, 99:1:0.1, *v:v:v*). 

For studying the pharmacodynamic and pharmacokinetic properties of the malaria drug lumefantrine (Supplementary Materials Figure S20), its enantiomer ratio had to be evaluated and quantified. For this purpose, Siqueira et al. [51] employed a commercial chiral column, namely, Chiralpak**^®^** AD-H (150 × 4.6 mm, i.d., 5 µM silica gel, Table 2). The optimal chromatographic conditions for the baseline ES of lumefantrine were as follows: 25 °C operative temperature, a mixture of *n*-hexane and isopropanol (97:3, *v:v*) as a normal mobile phase, flow rate of 1.0 mL/min, and UV detection at 335 nm. 

A method for the isolation of (*R*)-5-[1-(4-nitrobenzylsulfonyloxy)-ethyl]-5-(pyridine-2-yl)-[1,3,4]-thiadiazole (Supplementary Materials Figure S21), as a precursor in the synthesis of the antibiotic nafithromycin, was developed by Rane et al. [52], who used the commercial chiral column Chiralpak**^®^** IA (250 × 4.6 mm, i.d., 5 µM silica gel, Table 2), with a mixture of *n*-hexane and ethanol as the mobile phase. Thermodynamic investigations are also discussed herein. The limits of detection and quantification, as well as recovery of the undesired (*S*)-enantiomer, were 0.11, 0.35 μg/mL, and 104.52–105.83%, respectively.

In the area of pesticide analysis, a commercial Chiralpak^®^ IG-3 chiral column (150 × 4.6 mm i.d., 3 µM silica gel, Table 2) was employed by Díaz Merino et al. [53], for the ES of 17 chiral pesticides in polar organic and reversed mobile-phase mode by HPLC. Neat isopropanol was a suitable mobile phase. A larger number of resolved chiral pesticides, and generally higher resolutions of these analytes, was achieved by the addition of trifluoroacetic acid and diethylamine to acetonitrile in reversed mobile-phase mode. Six chiral compounds, including imazapic, hydroprop, heptachlor, metolachlor, *O*-ethyl-*O*-(4-nitrophenyl)phenylphosphonothioate, and dioxathion, were enantio-separated for the first time with the Chiralpak^®^ IG-3 column, under either polar organic or reversed mobile-phase conditions.

D’Orazio et al. [54] performed a comparative study on the ES ability of amylose *tris*-(3-chloro-5-methylphenyl carbamate) immobilized onto silica, and tested several chiral neutral and weakly acidic analytes by nano-LC, HPLC, and CEC, with acetonitrile and aqueous acetonitrile as the eluents. The resolution of enantiomers was slightly better under HPLC conditions than with nano-LC. In terms of plate numbers and peak resolution, CEC offered clear advantages.

The ES of chiral pyrethroid insecticides (*α*-cypermethrin and tetramethrin, Supplementary Materials Figure S22) on Chiralpak^®^ IG, Chiralpak^®^ IA, Chiralpak^®^ IB, and Chiralpak^®^ IC, (250 × 4.6 mm i.d., 5 μm silica gel for all columns, Table 2) was explored by Zhao et al. [7]. The best option was a Chiralpak^®^ IG column, and the optimum mobile phase was composed of a mixture of acetonitrile/water (75:25, *v:v*). After being validated according to the recommendation of the European Union SANTE/11945/2015, the developed method was applied to the stereoselective analysis of tetramethrin and *α*-cypermethrin in food products.

Four commercial chiral columns, Chiralpak^®^ IG-3 (250 × 4.6 mm, i.d., 3 μm silica gel), Chiralpak^®^ IG (250 × 10 mm, i.d., 5 μm silica gel), Chiralpak^®^ IA-3 (250 × 4.6 mm, i.d., 3 μm silica gel), and Chiralpak^®^ IA (250 × 10 mm, i.d., 5 μm silica gel) (Table 2), were chosen by Panella et al. [55], to study the effects of temperature and mobile-phase composition on the ES of the drug carvedilol (Supplementary Materials Figure S23), by HPLC. The enantiomers of carvedilol were baseline-separated on amylose *tris*-(3-chloro-5-methylphenyl carbamate) and amylose *tris*-(3,5-dimethylphenyl carbamate) as CSPs, under normal-phase, polar organic, and reversed-phase conditions. However, for semi-preparative applications, a non-standard ethyl acetate-based mobile phase was recommended.

A combination of chiral liquid chromatography and MS, in an attempt to increase the selective and sensitive qualification and quantification of ephedrine and pseudoephedrine as illicit drugs (Supplementary Materials Figure S24), has been reported by Karakka Kal et al. [56]. A large set of different chiral columns was used in this study, which are commercially available as Chiralpak^®^ OD-H, Chiralpak^®^ OJ-H, Chiralpak^®^ IC, Lux^®^ Cellulose-1, Lux^®^ Cellulose-2, Lux^®^ i-Cellulose-5 (Table 1), Chiralpak^®^ AD-H, Chiralpak^®^ IA, YMC CHIRAL ART Amylose-C, Lux^®^ Amylose-1, Lux^®^ i-Amylose-1, and Lux^®^ Amylose-2 (Table 2). Lux^®^ i-Amylose-1 had very broad and balanced enantio-recognition properties towards ephedrine analogs.

In the method development of ES of beta-blockers, alpha-blockers, anti-inflammatory compounds, sedative hypnotics, antiarrhythmic drugs, norepinephrine–dopamine reuptake inhibitors, catecholamines, antihistamines, flavonoids, amino acids, and antifungals, the two commercial chiral columns Chiralpak**^®^** IG-U and Chiralpak**^®^** ID-U (50 × 3.0 mm, i.d., 1.6 μm silica gel for all the columns, Table 2) have been used in normal-phase, polar organic and reversed-phase mode HPLC, by Ibrahim and Ghanem [57]. In the case of non-polar solvents (*n*-hexane and *n*-heptane) containing a polar alcohol modifier (methanol, ethanol, 2-propanol, and *n*-butanol), ethanol was the best option for the baseline ES of the analytes on the two CSPs. Regarding the non-standard solvent systems of methyl *tert*-butyl ether with different organic modifiers (ethanol, acetonitrile, and 1,4-dioxane), the highest enantioselectivity was obtained on Chiralpak**^®^** IG-U, with a mixture of methyl *tert*-butyl ether and 1,4-dioxane (90:10, *v:v*) as the mobile phase. However, this type of eluent did not show sufficient ES on Chiralpak**^®^** ID-U. Also, neat organic solvents did not enhance enantioselectivity in comparison to aqueous mixtures of them. The ES efficiency of Chiralpak**^®^** IG-U was higher than that of Chiralpak**^®^** ID-U, concerning the number of separated analytes. The authors also noticed that this was the first report of the partial separation of amino acids on amylose-based sub-2-micron columns.

Seven commercial chiral columns, namely, Chiralpak**^®^** IA, Chiralpak**^®^** ID, Chiralpak**^®^** IE, Chiralpak**^®^** IF, Chiralpak**^®^** IG, Chiralpak**^®^** IB, and Chiralpak**^®^** IC, (250 × 4.6 mm, i.d., 5 μm silica gel for all the columns, Table 2) were employed in method development, by Bajtai et al. [58], for the ES of new chiral amino-compounds, stereoisomers of *α*-hydroxynaphthyl-*ß*-carboline, benz[*d*]azepine and benz[*c*]azepine analogs, and *N-α*-hydroxynaphthylbenzyl-substituted isoquinolines (Supplementary Materials Figure S25), under normal mobile-phase conditions, applying HPLC and SFC.

The seven CSP polysaccharide derivatives were shown to have partially complementary characters, which eventually resulted in successful baseline separation of the targeted analytes. An increased share of the apolar component in the eluent (*n*-hexane or CO_2_) increased the retention, but only a minor change in enantiomeric discrimination was observed. The use of different alcohols as polar modifiers indicated that bulky alcohols with longer chains increased retention, while their effects on the resolution were also moderate. Two polar modifiers (2-propanol and methanol) were advantageous for normal-phase HPLC and SFC. Studies regarding the relationship of the structure and retention of both the analytes and CSs established that chiral recognition was induced by the steric fit, hydrogen bonding, and *π*–*π* interactions. Also, enthalpy control was observed for most of the analytes, as well as differences in ES of the above-mentioned analytes between normal-phase HPLC and SFC. 

In an extensive study, 36 racemates of natural phytoalexins were resolved on commercial chiral columns, namely, Chiralpak**^®^** IA, Chiralpak**^®^** IB, and Chiralpak**^®^** IC (250 × 4.6 mm, i.d., 5 μm silica gel for all the columns, Table 2), under normal-phase mode by HPLC, as reported by Petrovaj et al. [59]. Chiralpak**^®^** IA and Chiralpak**^®^** IB exhibited better chiral recognition ability towards phytoalexin enantiomers than Chiralpak**^®^** IC. Enantiomer separation, in the cases of Chiralpak**^®^** IA, Chiralpak**^®^** IB, and Chiralpak**^®^** IC, was generally controlled by enthalpy, while the entropic effects were more pronounced in the case of Chiralpak**^®^** IC. The developed method was shown to be more efficient, faster, and to have better chiral recognition than previously reported methods. 

Enantiomeric phytoalexins were separated on amylose derivative CSs in SFC, as shown by Kozlov et al. [60]. Commercial chiral columns, Trefoil AMY1 (Table 2) and Trefoil CEL1 (Table 1) (Waters Corp., 50 × 3.0 mm, i.d., 2.5 μm silica gel for all the columns), were employed to separate 27 indole phytoalexins as potential anticancer and antimicrobial drugs, under SFC conditions. The amylose derivative CSs gave a higher number of baseline-separated compounds than the respective cellulose derivative CSs.

Another approach in chiral SFC is the combination with MS as a detection method. A method for ES and quantification of nine non-steroidal anti-inflammatory drugs, including ibuprofen, indoprofen, pranoprofen, flurbiprofen, ketoprofen, carprofen, naproxen, loxoprofen, and etodolac (Supplementary Materials Figure S26), extracted from fish tissues, on polysaccharide derivatives as CSs, by SFC coupled to tandem MS, was developed by Li et al. [61]. The ES efficiency of the four commercially available chiral columns Chiralpak^®^ IA, IB, IC, and ID (250 × 4.6 mm, i.d, 5 µM silica gel for all the columns, see Table 2) towards these analytes was compared. Among the investigated columns, Chiralpak^®^ ID showed the highest enantioselectivity towards seven of the nine chiral drugs. The resolution values of ibuprofen, indoprofen, pranoprofen, flurbiprofen, ketoprofen, naproxen and carprofen were *R_s_* = 0.66, 3.56, 6.04, 7.17, 1.69, 2.29 and 3.29, respectively. The technique was applied to the analysis of real fish samples, providing a reference method for the analysis of these drug enantiomers in food or environmental samples.

## 4. Chitosan-Based CSPs

Chitosan 3,6-*bis*-(phenyl carbamate)-based CSs, with different substituents at C2, were studied in 2019 and 2020. The effects of the different molecular weights of the chitosan obtained from different sources (shrimp shell and crab shell) on the ES performance of the derivatives, were studied by Zhang et al. [62]. Chitosan was *N*-isobutyrylated to produce isopropylcarbonyl chitosans, which were then derivatized with 4-methylphenyl isocyanate to obtain chitosan 3,6-*bis*-(4-methylphenyl carbamate)-2-(isobutyrylamide) as CSs (Supplementary Materials Figure S27). Chitosans with different molecular weights were prepared from the same source material, by alkaline hydrolysis. The CSs with a higher molecular weight exhibited better ES efficiency than the ones with a comparatively low molecular weight. Chitosan that was extracted from shrimp shells exhibited better chromatographic performance than the one obtained from crab shells at a similar molecular weight, even though the authors concluded that the overall difference in performance was not very significant.

**Figure 5:**
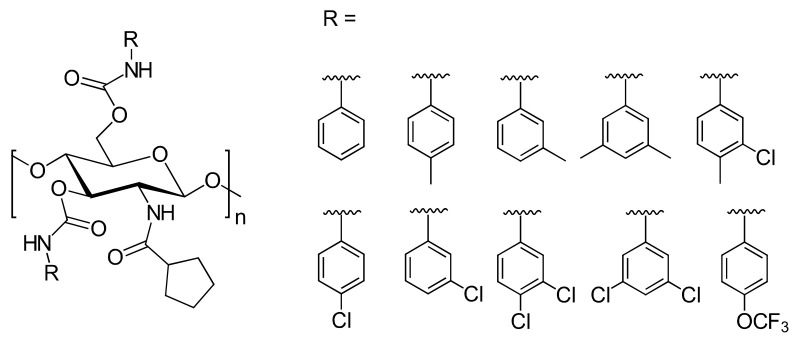
Chemical structures of different chitosan 3,6-*bis*-(aryl carbamate)-2-(*N*-cyclopentylcarbonyl) derivative-based CSs synthesized by Fu et al. [63].

In another approach, *N*-cyclopentylcarbonyl chitosan derivatives, comprising ten 3,6-*bis*-(arylcarbamate)-2-(cyclopentylformamide) derivatives with different substituents at the phenyl moiety, were synthesized by Fu et al. [63] (Figure 5). Tröger’s base, 2-phenylchroman-4-one, 1-(2-napthyl)-ethanol, methyl phenyl sulfoxide, 1-phenylethanol, mephobarbital, 4-phenyloxazolidin-2-one, 1-(1-phenylethyl)-3-(3-p-tolyl)urea, 1-(2,4-dichlorophenyl)-2-(1*H*-imidazol-1-yl)ethanol, 4-methyl-*N*-(1-phenylethyl)benzamide, benzoin, 1-(1-(4-methoxyphenyl)ethyl)-3-phenylurea, aminoglutethimide, glutethimide, citalopram hydrobromide, efavirenz, *N*-(1-(4-methoxyphenyl)ethyl)-3,5-dinitobenzamide, omeprazole sodium, 4-(4-(dimethylamino)-1-(4-fluorophenyl)-1-hydroxybutyl)-3-(hydroxymethyl)benzonitrile, and voriconazole were employed as the analytes to evaluate the ES performance of the chitosan-based CSs. *N*-Cyclopentylcarbonyl chitosan derivatives exhibited a higher ES capacity compared to the respective cyclopropylcarbonyl, cyclobutylcarbonyl, and cyclohexylcarbonyl chitosans. Methyl or halogen substituents at the phenyl carbamate moieties were shown to significantly contribute to the ES performance of *N*-cyclopentylcarbonyl chitosan derivatives; CSPs with halogen substituents at the aromatic ring performed better than the methyl-substituted ones. The chitosan derivative with a 3,4-dichlorophenyl substituent baseline separated the investigated chiral analytes. Also, the CSs were highly tolerant to common mobile phases, such as acetone or ethyl acetate. Therefore, the developed CSPs were further investigated regarding ES performance, by extending the mobile-phase systems.

Chitosan 3,6-*bis*-(phenyl carbamate)-2-(cyclobutylcarbamoyl) derivatives (Supplementary Materials Figure S28) were synthesized with well-deacetylated chitosan and halogen-substituted phenyl isocyanate, and were applied as potential CSs by Zhang et al. [64]. After coating onto modified silica gel, the ES efficiency of the developed columns was evaluated by HPLC and compared to the commercial chiral column material Chiralcel^®^ OD-H (250 × 4.6 mm, i.d., 5 μm silica gel). All the chitosan 3,6-*bis*-(phenyl carbamate)-2-(cyclobutylcarbamoyl) derivatives provided a satisfactory ES efficiency, with the 3,4-dichlorophenyl-substituted derivatives showing the highest chiral recognition ability. It is noteworthy that all of the prepared CSPs exhibited excellent tolerance to “unusual solvents”, and some of them could even be used in pure tetrahydrofuran. The developed CSPs were therefore considered to be promising candidates for enhancing ES in non-standard mobile phases.

Four derivatives of chitosan 3,6-*bis*-(chlorophenyl carbamate)-2-isobutylurea were studied by Zhang et al. [65] (Supplementary Materials Figure S29). The compounds were stable in pure ethyl acetate, pure acetone, or *n*-hexane/tetrahydrofuran mixtures, with promising ES under HPLC conditions. When comparing to coated amylose cellulose derivatives as CSPs, the developed chitosan derivatives exhibited a better ES performance.

A new approach in electrochemical chiral recognition, based on chitosan as CS, was studied by Yang et al. [66]. Graphene was functionalized with 3,4,9,10-perylene tetracarboxylic acid, and then an amidation reaction was employed to anchor chitosan onto graphene. The chiral nature of chitosan, and the electrochemical behavior of the functionalized graphene, provided a unique hybrid character. A glassy carbon electrode, modified with the developed composite, was then applied as a stereoselective chiral sensor, for the electrochemical chiral recognition of tryptophan enantiomers. The sensor was more sensitive towards l-tryptophan than towards the d-counterpart, and had high accuracy and linear response in the range of 1–10 mM of tryptophan, with detection limits of 1.2 μM for l-tryptophan and 3.0 μM for d-tryptophan.

Molecular imprinting is also a common method for the preparation of enantioselective materials. Xiao et al. have prepared chitosan resins that are imprinted with *S*-mandelic acid, by cross-linking chitosan with glutaraldehyde in 2% acetic acid solution, for ES of racemic mandelic acid in an aqueous medium [67]. Employing a buffer solution (100 mM *Tris*-H_3_PO_4_, pH = 3.5), the enantiomer excess of *R*-mandelic acid in the supernatant was 79% after 40 min of adsorption time. The imprinted chitosan resin possessed generally higher adsorption capacities toward *S*-mandelic and *R*-mandelic acid (29.5 vs. 2.03 mg/g) compared to non-imprinted chitosan (2.10 vs. 2.08 mg/g).

## 5. β-Cyclodextrin-Based CSPs

### 5.1. Chiral Selector Development

A variety of new CSs based on β-cyclodextrin have been introduced in the last two years. Cyclodextrins (CDs) are characterized by a variety of chiral centers, in which an extraordinary feature of CDs is their chiral, rather lipophilic cavity. This cavity is ideally suited for the incorporation of lipophilic structural motifs of organic (chiral) compounds, for example, aromatic units. Due to the large number of primary and secondary hydroxy groups, a multitude of options for chemical derivatization, for introducing interaction sites and chemical anchors for immobilization onto, e.g., silica carriers or nanomaterials, are available.

Tethering β-cyclodextrin derivatives to the support matrix, to enhance chiral recognition ability, is a new approach in β-cyclodextrin-based CSs [68,69,70]. A more detailed insight into the mechanism of enantiodifferentiation with cyclodextrins is referred to in Ji et al. [71], Wei et al. [72], and Dai and Wu et al. [73].

**Figure 6:**
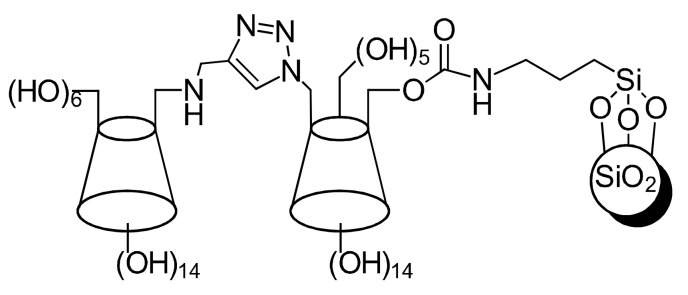
Chemical structure of a triazole-bridged *bis*-(β-cyclodextrin) bonded on silica gel as CSP as used in the study of Shuang et al. [68].

An efficient, high-yield “*click chemistry*” reaction between 6-azido-β-cyclodextrin and 6-propynylamino-β-cyclodextrin was performed, to produce a triazole-bridged *bis*-(β-cyclodextrin), which was then immobilized onto silica gel in an attempt to employ β-cyclodextrin as a material for ES of chiral pesticides and drugs, as performed by Shuang et al. [68] (Figure 6). 

The ES ability of the triazole-bridged *bis*-(β-cyclodextrin) was then evaluated by HPLC. In reversed mobile-phase mode, the CS successfully separated nine triazole-type analytes, eight flavanones, and eight *N*-dansyl-modified amino acids. Especially, the resolutions of hexaconazole, 2′-hydroxyflavanone, and dansyl-d,l-tyrosine were as high as 2.49, 5.40, and 3.25, respectively, within 30 min of the analysis time. ESs of ten *β*-blockers on the triazole-bridged *bis*-(β-cyclodextrin) stationary phases had to be carried out in polar organic mode. The benefits from the synergistic inclusion ability of the two adjacent cyclodextrin cavities resulted in higher ES ability in comparison to native β-cyclodextrin. The CSP showed high chemical stability and good chromatographic reproducibility, and was therefore suggested as a promising candidate for the ES of drugs and food analysis.

Shuang et al. have continued employing 4,4′-stilbene dicarboxylic acid as a linker to prepare bridged β-cyclodextrin as CS [69]. The reaction between 4,4′-stilbene dicarboxylic acid and 6-deoxy-6-amino-β-cyclodextrin was performed, to produce a stilbene diamido-bridged *bis*-(β-cyclodextrin), which was then immobilized onto the surface of modified mesoporous silica gel (Supplementary Materials Figure S30). The ES of 23 racemic drugs and pesticides was tested on this phase, in reversed-phase or polar organic mode. All the analytes were baseline-resolved with high resolutions, in the range of 1.51–5.15, within an analysis time of 25 min. For example, the *R_s_* values of flavanone and imazalil were up to 5.15 and 4.38, respectively. Five pairs of triazole-type pesticide enantiomers were simultaneously separated on the developed CS within 30 min. A comparison on the enantioselectivity and diastereoselectivity of native β-cyclodextrin and the stilbene diamido-bridged *bis*-(β-cyclodextrin) was performed, in which the latter showed better chromatographic performance. In particular, trimeprazine, praziquantel, flavanone, and imazalil could not be separated on native β-cyclodextrin as CSP, but were baseline-resolved on the bridged derivative, with high resolutions. The authors suggested that the bridging linker of the selector provides a well-organized “*pseudocavity*”, which synergistically encapsulates and complexes bulkier analytes, while only a small cavity of 0.65 nm is found in native β-cyclodextrin. 

A similar class of CSPs, based on tethered β-cyclodextrin, was reported by Shuang et al. [70]. The 6-deoxy-6-amino-β-cyclodextrin was amidated with ethylenediaminetetraacetic dianhydride, to produce an ethylenediamine dicarboxyethyl diacetamido-bridged *bis*-(β-cyclodextrin), which was then immobilized onto the surface of modified silica gel as CSP (Supplementary Materials Figure S31). The ES performance of the CSP was evaluated with 28 racemates (eight flavanones, eight blockers, five dansyl amino acids, three amino acids, and four common drugs) as analytes, in reversed-phase or polar organic HPLC. All of the analytes were separated on the developed CSP, and 20 of them were even baseline-separated. Especially, 2′-hydroxyflavanone and arotinolol had resolution values up to 4.35 and 2.05, respectively, within 30 min of the analysis time, while with native β-cyclodextrin, it was only possible to separate 11 analytes, with comparatively low resolutions (*R_s_* = 0.55–1.69). It is noteworthy that the newly developed CSP was able to directly separate d,l-amino acids with a mobile phase containing Cu^2+^ ions, due to the complexation of the EDTA-based bridging linker. 

A chiral selector, based on heptakis(6-*O*-methyl)-β-cyclodextrin, was synthesized in five steps, under heterogeneous, chromatography-free, and up-scalable conditions, by Balint et al. [74]. The compound showed promising properties towards the separation of the drug methylenedioxypyrovalerone, as a model analyte in capillary electrophoresis.

A chiral column material, with fully derivatized 4-chlorophenyl carbamate-β-cyclodextrin immobilized onto silica gel as CSP, was introduced by Sun et al. [75], for the ES of twelve azole-type antifungal agents, five proton pump inhibitor drugs, and five dihydropyridine calcium antagonists, used in both reversed mobile-phase and normal mobile-phase HPLC. The ES of all the analytes was achieved; the resolution of the twelve azole antifungal agents was excellent. For example, the *α* and *R_s_* values of voriconazole were 15.41 and 16.80, respectively. The normal-phase elution mode exhibited higher enantiomeric selectivity than the reversed mobile-phase mode. The authors indicated inclusion, hydrogen bonding, and *π*–*π* interaction as the main driving interactions of the ES process in the normal-phase mode. The 4-chlorophenyl carbamate groups are beneficial for chiral recognition, since all the analytes, except ilaprazole, were better separated on the newly developed CS than on a 3,5-dimethylphenyl carbamate-β-cyclodextrin selector. The analytical-scale column material was scaled up to a semi-preparative scale for single enantiomer isolation, and was shown to be stable and to give reproducible separations.

Zhao et al. synthesized carboxymethyl-6-(4-methoxybenzylamino)-β-cyclodextrin as CS for the ES of chiral drugs (chlorpheniramine, brompheniramine, pheniramine, homatropine, homatropine methyl, clorprenaline, terbutaline, procaterol, tulobuterol, cycloclenbuterol, propranolol, pindolol, and ofloxacin, see Supplementary Materials Figure S32), by capillary electrophoresis [76]. A fused-silica capillary (50 cm × 50 µM, i.d., 40 cm effective length), rinsed with the β-cyclodextrin derivative, was employed for ES in 30 mM phosphate buffer at 20 kV. Eleven of the analytes were baseline-separated and two (tulobuterol and pindolol) were partially separated. A novel CSP, based on β-cyclodextrin for HPLC, was developed by Ren et al. [77], employing copolymerization of thermoresponsive poly(*N*-isopropylacrylamide) and β-cyclodextrin, and subsequent grafting onto silica beads. The thermoresponsive modulation of the developed CSP, regarding ES, was investigated, with racemates of 1-phenyl-1-propanol, styrene oxide, 2-phenylpropionic acid, ibuprofen, and labetalol hydrochloride as the analytes, and all of them were separated. The newly developed CSP changed from a hydrophilic character to a hydrophobic character in the temperature range of 10–60 °C. Thus, by regulating the operation temperature, one could modulate the types of interaction. Also, positional isomers, alkylbenzenes, flavonoids, and benzoic acids were baseline-separated on the developed CSP in reversed mobile-phase mode HPLC. The CSP was shown to be promising for the ES of both hydrophobic and hydrophilic chiral compounds. 

In another approach, Ke et al. [78] employed β-cyclodextrin as a starting material for preparing a chiral thin-film composite membrane. Cyclodextrin was allowed to react with *p*-toluenesulfonyl chloride to obtain intermediate 6-*O*-monotosyl-β-cyclodextrin. Via the introduction of this excellent leaving group, mono-6-diethylamino-β-cyclodextrin as CS was obtained after reaction with diethylamine. The CS was interfacially crosslinked with trimesoyl chloride on the surface of a commercial cellulose acetate membrane. Chiral drugs, such as warfarin, ibuprofen, nefopam, ketoprofen, and tryptophan (Supplementary Materials Figure S33), were used to evaluate the ES performance of the membrane. The enantiomeric excesses of warfarin, ibuprofen, and tryptophan were about 9.3%, 3.8%, and 27.2%, respectively.

### 5.2. Preparation Developments and Method Development

A chiral column with benzylureido-β-cyclodextrin bound to silica gel as CSP (Supplementary Materials Figure S34), via a “*thiol-ene click addition*” reaction, was prepared by Li et al. [79], to enantioseparate phenylmercapturic and benzylmercapturic acid (Supplementary Materials Figure S35). The two analytes were well separated within 30 min (*R_s_* = 2.25 and 2.14, respectively) in reversed-phase mode UHPLC. Also, a new method for qualifying and quantifying phenylmercapturic and benzylmercapturic acid enantiomers, by liquid chromatography coupled with MS (negative ion multiple reaction monitoring), applying D_2_-labeled phenylmercapturic acid as an internal standard, was elaborated.

Recently, graphene quantum dots have also been discovered as a carrier component to prepare CSPs. A big advantage of such nanomaterials is their large specific surface area. The higher the surface area, the more interaction sites are possible, eventually increasing the potential chiral recognition, by stronger retention of one enantiomer. In this context, it should also be mentioned that either intrinsically chiral nanomaterials (e.g., chiral nanotubes), but also achiral nanomaterials, which have been modified with chiral molecules, are used. Wu et al., for example, have immobilized β-cyclodextrin onto graphene quantum dots, to be used as CSPs for ES, under HPLC conditions [80]. The quantum dots indeed enhanced the ES performance of unmodified β-cyclodextrin, a β-cyclodextrin 3,5-dimethylphenyl carbamate derivative, and cellulose *tris*-(3,5-dimethylphenyl carbamate), in comparison to their counterparts, by indirectly increasing the interaction of analyte and CS, which was indicated by a molecular simulation study. Thus, graphene quantum dots were shown to be promising as chromatographic support for chiral separations.

A chiral liquid chromatography column, prepared by coating hydroxypropyl-β-cyclodextrin onto a reversed-phase porous silicon pillar array column, was developed by Naghdi et al. [81]. The ES efficiency of the developed column was evaluated with mixtures of non-steroidal anti-inflammatory drugs as the analytes, under nano-LC conditions. The CSP exhibited good chromatographic properties, and the chromatographic efficiency of the column was maintained by sufficient flushing and repeating of the loading procedure. The column was also able to separate diastereomeric compounds. The potential of CSPs, with regard to the separation of diastereomers, structural isomers, homologous and/or structurally unrelated compounds, or even achiral compounds, is far from being exhausted. In the future, the focus will also move more and more towards chiral × chiral and chiral × achiral 2D (LC) separations.

Besides HPLC, capillary chromatography has also emerged as an analytical method using β-cyclodextrin as CS. New techniques to immobilize β-cyclodextrin, and the development of a new inert carrier have been investigated in recent years. A sol-gel technique was exploited by Jiang et al. [82], to prepare an open tubular capillary electrochromatography column with coated β-cyclodextrin gold nanoparticles on the inner wall, as CSP. Generally, the use of beta-CD functionalized Au NPs, in combination with CEC, is predominant in this field, because the technology for the preparation of functionalized Au NPs is a well-known technique. The capillary column exhibited good stability and repeatability for ES purposes, due to the three-dimensional network obtained by the sol-gel process, and the resulting strong chemical bonds between the stationary phase and the surface of the capillary column. The ESs of chlorphenamine, brompheniramine, pheniramine, and zopiclone were better on the developed column than on the pure sol-gel matrix capillary column, because the gold nanoparticles as electro-chromatographic support enhanced the phase ratio of the open tubular capillary column. The relative standard deviation of the ES for the migration time was less than 0.89% over five runs and 2.9% from column to column.

In another approach, gold nanoparticles were taken advantage of by Zhou et al., to prepare CSPs [83]. A multilayer carrier was prepared, by coating polydopamine and gold nanoparticles onto the surface of a bare fused-silica capillary, before immobilizing sulfated β-cyclodextrin as CS. Ten chiral drugs (salbutamol, terbutaline, trantinterol, tulobuterol, clorprenaline, pheniramine, chlorpheniramine, brompheniramine, isoprenaline, and tolterodine) were employed, to evaluate the ES performance of the capillary electrochromatography column. These analytes were baseline-separated, with resolution (*R_s_*) values between 1.76 and 3.25. Also, the repeatability of the developed column was studied, with the relative run-to-run, day-to-day and column-to-column standard deviations being lower than 5.7%.

In a similar study, Wang et al. introduced quantum dot-functionalized β-cyclodextrin and its derivatives as CSPs for ES, in a combined system of capillary electrophoresis, with laser-induced fluorescence detection [84]. Because of the chirality of β-cyclodextrin and the fluorescence property of graphene quantum dots, the combination of capillary electrophoresis with laser-induced fluorescence detection was successfully applied to separate d,l-tryptophan, d,l-phenylalanine, d,l-tyrosine, epicatechin, catechin, and ritodrine hydrochloride. Furthermore, this developed method was successfully applied to analyze the active components in the Chinese herb catechu (catechin and epicatechin), and the recoveries were in the range of 92–108%.

Although a large variety of chiral compounds, with different chemical structures, can be separated efficiently by cyclodextrin-modified nanoparticle-based systems, detailed in-depth studies are often missing, or are only sparsely explained. In the above-mentioned publications, however, it is evident that the linking of nanoparticles with cyclodextrins can have both a positive as well as a negative influence on the separation parameters. Detailed studies on this are certainly important for future developments in this area.

A mixed Ni/graphene electrode as a potential inert carrier was applied by Chen et al. [85]. After plasma-enhanced chemical vapor deposition, the Ni/graphene hybrid electrode was surface-coated with β-cyclodextrin, to prepare a CSP. The ES performance was evaluated with d,l-phenylalanine, which was separated with a 0.09 V peak shift in differential pulse voltammetry tests. Also, the wide linear range for the detection of l-phenylalanine by the developed CSP was an advantage, as were the good storage and working stability.

A metal–organic framework (MOF) of the Cu_3_(1,3,5-benzenetricarboxylic acid)_2_ type as chromatographic support in capillary electrochromatography was studied by Sun et al. [86]. After modification with carboxymethyl-β-cyclodextrin, the developed CSP was evaluated with the drugs propranolol, esmolol, metoprolol, amlodipine, and sotalol. In comparison to a conventional capillary (fused-silica), the resolutions of these analytes were significantly improved.

A monolithic capillary column, functionalized with hydroxypropyl-β-cyclodextrin for ES of racemic anticholinergic drugs, β-adrenergic drugs, meptazinol, and its intermediates, was developed by Deng et al. [87]. The developed column has been prepared in one pot, with two sequential reactions. All of the tested analytes were baseline-separated on the developed chiral column material; especially, an excellent separation towards four flavanone glycoside epimers was observed. β-Cyclodextrin-functionalized magnetic particles on the inner walls of the capillary columns, for enhancing the ES after applying an external magnetic field, was exploited by different research groups, inter alia Yang et al. [88]. After functionalization with magnetic nanoparticles, β-cyclodextrin, and mono-6-deoxy-6-(1-methylimidazolium)-β-cyclodextrin were coated onto an inner wall of a capillary column. The ES efficiencies of these CSPs were verified with six amino acids (alanine, leucine, isoleucine, valine, methionine, and glutamic acid). The developed capillaries showed excellent chiral recognition ability, and also good reproducibility and durability. 

Magnetic microparticles were also taken into consideration in the work by Sun et al., who prepared capillary electrochromatography columns for ES [89]. Carboxymethyl-β-cyclodextrin functionalized with magnetic microparticles was coated onto the inner walls of a capillary column, and the resulting CSP was evaluated in an open tubular capillary electrochromatography system, with several racemic drugs. A new type of capillary column was reported by Sun et al. [90]. The authors coated glycidyl methacrylate as a film onto the inner capillary wall, onto which poly(glycidyl methacrylate) nanoparticles were then immobilized. By a ring-opening reaction, ethanediamine-β-cyclodextrin was immobilized onto the nanoparticles as CSP. The resulting capillary column was compared with a reference (a single layer of β-cyclodextrin with glycidyl methacrylate or β-cyclodextrin with poly(glycidyl methacrylate) nanoparticles), regarding the ES of the drugs propranolol, amlodipine, and metoprolol as the racemic analytes. The ES performance of the newly developed capillary column was significantly enhanced, and very satisfying resolution values (propranolol: 1.27, metoprolol: 1.01, and amlodipine: 2.93) were obtained. Therefore, the authors suggested that this type of CSP was also likely to have a great potential in the ES of other enantiomers, such as amino acids and biogenic amines.

Glutaraldehyde as a linker was exploited by Khatri et al. [91], to immobilize β-cyclodextrin onto poly (vinyl alcohol), to prepare a CSP to be used in thin-layer chromato-graphy (TLC). The ES performance of this planar phase was evaluated with enantiomeric pairs of histidine and serine. The amino acids were detected by spraying with ninhydrin solution as the staining reagent. Among the various solvent systems employed, a mixture of ethanol/butanol/ethyl acetate/water/acetone (4:5:5:0.5:1.5, *v:v*) as the mobile phase gave good separation of serine enantiomers (*R_s_* = 1.6), and ES of histidine with a resolution of 1.4 was possible with the mobile phase of ethanol/butanol/ethyl acetate/water/acetone (4:5:4.5:0.5:1.5, *v:v*). This proved the prepared CSP to be efficient in ES of the selected amino acids, so that an extension of the study to physiological samples was envisioned.

Coating sulfobutylether-cyclodextrin with different degrees of substitutions (4, 6.3, and 10) onto strong anion exchange stationary phases (silica-based quaternary ammonium-bound sorbent), for the preparation of CSPs, was an approach reported by Folprechtová et al. [92]. The ES potential and stability of the developed CSP was evaluated with the chiral drugs oxazepam, lorazepam, promethazine, thioridazine, carprofen, fenoprofen, flurbiprofen, indoprofen, as well as 6-hydroxyflavanone, 7-hydroxyflavanone, *tert*-butyloxycarbonyl-tryptophan, Tröger’s base, propranolol, and 5-fluor-tryptophan, employing reversed phase/polar organic mode HPLC (methanol/formic acid, pH 2.10, and methanol/10 mM ammonium acetate buffer, pH 4.00, in various volume ratios). The CSPs with degrees of substitution of 4, 6.3, and 10 were able to separate 14, 11, and 8 of the analytes, respectively. Nearly all the basic and neutral analytes were baseline-resolved at a DS of four, while CSPs with a higher degree of substitution (DS = 10) were shown to work preferably for the ES of acidic analytes.

Sulfobutylether-β-cyclodextrin was also applied in ES of the anti-Parkinson drug rasagiline (Supplementary Materials Figure S36), under capillary electrophoresis conditions, by Szabó et al. [93]. The optimum conditions for a high resolution (*R_s_* = 3.5) were as follows: 50 mM glycine–HCl buffer pH 2.0, supplied with 30 mM sulfobutylether-β-cyclodextrin, 35 °C, applying 12 kV in reversed polarity, and –8 mbar pressure (vacuum), and an analysis time of 8 min. The developed method was reliable, linear, precise, and accurate for the determination of 0.15% *S*-enantiomer as a chiral impurity in an *R*-rasagiline sample, as well as for the quantification of the eutomer.

Fourteen different neutral and anionic cyclodextrins have been screened in a study by Papp et al., for ES of lansoprazole and rabeprazole (Supplementary Materials Figure S37), by capillary electrophoresis at pH 4.0 and 7.0 [94]. Both the analytes were baseline-separated on sulfobutylether-β-cyclodextrin, with a degree of substitution (DS) of 6.5 and 10 at neutral pH. A dual selector system containing sulfobutylether-β-cyclodextrin with DS 6.5 and native *γ*-cyclodextrin, proved to be the most adequate system for the separations. The separation conditions were optimized by a central composite design, with a lansoprazole resolution value of *R_s_* = 2.91 and rabeprazole resolution value of *R_s_* = 2.53. The optimized methods were validated and proved to be reliable, linear, precise, and accurate for the determination of 0.15% distomer as a chiral impurity in dexlansoprazole and dexrabeprazole samples.

Sulfated β-cyclodextrin was used as a chiral mobile-phase additive for ES of the anti-depressant drug milnacipran (Supplementary Materials Figure S38), under reversed-phase HPLC with a core–shell Kinetex C_8_ column (150 × 4.6 mm, 5 µM), by Pathak et al. [95]. A mobile phase consisting of 18:82 (*v:v*) acetonitrile/10 mM aqueous sodium dihydrogen phosphate buffer pH 3.0, containing 10 mM sulfated β-cyclodextrin, was ideal for resolving the milnacipran enantiomers. The method was also applied to commercial samples, after validating it according to the International Council for Harmonisation of Technical Requirements for Pharmaceuticals for Human Use. The authors concluded that the use of the core–shell-type column material would reduce both the analysis time and the amount of CS, in comparison to a fully porous column material.

Different types of β-cyclodextrin (native β-cyclodextrin, acetyl-β-cyclodextrin, 2-hydroxypropyl-β-cyclodextrin, and carboxymethyl-β-cyclodextrin) were screened for ES of 61 cathinone and pyrovalerone derivatives, used as psychoactive substances, under capillary electrophoresis conditions, by Hagele et al. [96]. Overall, 58 out of 61 analytes were partially or fully baseline-separated within 40 min, by at least one of the four CSs, using 10 mM β-cyclodextrin derivatives in a 10 mM sodium phosphate buffer (pH 2.5). The resolution values ranged from 0.3 to 6.2. Furthermore, the developed method was found to have a wide range of applications, including simultaneous ES, enantiomeric purity checks, and analysis of positional isomers. 

Shi et al. have employed a commercial chiral capillary GC column Astec^®^ CHIRALDEX^®^ B-PM (50 m × 0.25 mm × 0.12 µM, L × i.d. × d_f_), coated with 2,3,6-tri-*O*-methyl-β-cyclodextrin, and six in-house prepared chiral columns (20 m × 0.25 mm, i.d., 0.31 μm film thickness), coated with 2,3-di-*O*-pentyl-6-*O*-propyl-β-cyclodextrin, 2,6-di-*O*-pentyl-3-*O*-butyryl-β-cyclodextrin, 2,3-di-*O*-pentyl-6-*O*-octanoyl-β-cyclodextrin, 2,6-di-*O*-benzyl-3-*O*-heptanoyl-β-cyclodextrin, 2,3,6-tri-*O*-valeryl-β-cyclodextrin, and 2,3,6-tri-*O*-octanoyl-β-cyclodextrin for ES of eight enantiomer pairs of alkyl 2-bromopropionates and five enantiomer pairs of cycloalkyl 2-bromopropionates, under GC conditions. These analytes were either raw materials or intermediates for the synthesis of chiral pesticides and drugs [97].

The Dubsky’s model for CE enantioseparations was applied by Casado et al. [98], to test a mixture of chiral selectors, (2-hydroxypropyl)-β-cyclodextrin, and heptakis (2,3,6-tri-*O*-methyl)-β-cyclodextrin, for ES of the following six phenoxyacid herbicides: fenoprop, mecoprop, dichlorprop, 2-(4-chlorophenoxy)propionic acid, 2-(3-chlorophenoxy)propionic acid, and 2-phenoxypropionic acid (Supplementary Materials Figure S39). A dual system, combining the two CSs, improved the results. The resolution increased significantly, from 1.2 to 4.2, by the newly developed approach, which used 4 mM 2-hydroxypropyl-β-cyclodextrin and 16 mM heptakis (2,3,6-tri-*O*-methyl)-β-cyclodextrin as CSs, in comparison to the previous methods, and the analysis time was also shortened.

Four stereoisomers of sertraline (an antidepressant drug, see Supplementary Materials Figure S40) were successfully separated by Sun et al. [99], applying hydroxypropyl-β-cyclodextrin as CS in a liquid–liquid partition chromatography system. A biphasic solvent system, composed of *n*-hexane/0.20 M phosphate buffer solution at pH 7.6, together with 0.10 M hydroxypropyl-β-cyclodextrin (1:1, *v:v*), was used. 

A new method for ES of naproxen and warfarin, by micellar capillary electrophoretic chromatography, with hydroxypropyl-β-cyclodextrin as a CS and pyrrolidinium-based ionic liquid surfactants as the pseudostationary phase, was proposed by Yu et al. [100]. It provided better ES of the analytes than a comparable method based on conventional non-ionic liquid cationic surfactants.

## 6. Conclusions and Outlook

In conclusion, progressive methods for the ES of chiral compounds with commercial cellulose and amylose-based chiral columns have made tremendous progress, also, and in particular, within the past few years, which were covered in this overview. Despite many new and exciting developments in research labs, the number of commercially available—because reproducibly manufacturable—CSPs remains modest. Many analytical method developments are based on those established columns, and applications of them to new tasks of analytical or preparative ES. Here, the different types of columns, both commercial and newly developed, different CS materials, such as cellulose, amylose, chitosan, or cyclodextrin derivatives, and a variety of analytical approaches, have been summarized. Where available, data on the chiral recognition mechanisms on the particular chiral column material have been added. The developments with chitosan as the CS were minor in comparison to cellulose, amylose, and β-cyclodextrin. We suppose that further developments for CSP based on cellulose, amylose, chitosan, and β-cyclodextrin will be coupled to a better understanding of the fundamentals of chiral recognition mechanisms, which will allow replacing “let’s try and see” approaches and empirical optimization more and more by targeted developments and mechanism-based predictions. Another very promising aspect of recent and future developments is the application of CSPs, both in chiral × chiral as well as chiral × achiral 2D (liquid) chromatography. This will also lead to a great expansion of the analytical applications in the field of achiral chromatography. Given the developments in the field of core–shell materials, the effect of experimental influencing parameters, such as appropriate detector frequency and data-processing for high-speed separations, should also be considered.

## Figures and Tables

**Table 1:** Selection of cellulose derivative-based commercial chiral columns and chemical structure of the chiral selectors.

Commercial Name	Chiral Selector	Chemical Structure of the Chiral Selector
Chiralcel^®^ OD-H, Trefoil CEL1, Chiralcel^®^ OD-H, Chiralcel^®^ OD-RH, Chiralcel^®^ OD-3R, Chiralpak^®^ OD-H, Lux^®^ Cellulose-1	coated cellulose *tris*-(3,5-dimethylphenyl carbamate)	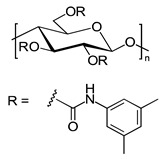
Chiralpak^®^ IB	immobilized cellulose *tris*-(3,5-dimethylphenyl carbamate)	
Chiralcel^®^ OZ-3R, Lux^®^ Cellulose-2	coated cellulose *tris*-(3-chloro-4-methylphenyl carbamate)	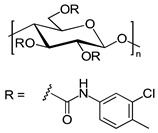
Chiralcel^®^ OJ-3R, Chiralcel^®^ OJ-H, Chiralcel^®^ OJ, Chiralpak^®^ OJ-H, Lux^®^ Cellulose-3	coated cellulose *tris*-(4-methyl benzoate)	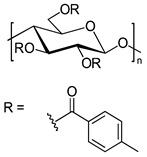
Lux^®^ Cellulose-4	coated cellulose *tris*-(3-methyl-4-chlorophenyl carbamate)	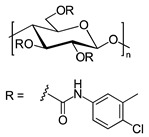
Chiralpak^®^ IC, Lux^®^ i-cellulose-5	immobilized cellulose *tris*-(3,5-dichlorophenyl carbamate)	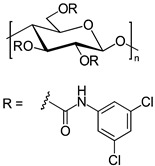

**Table 2:** Selection of cellulose derivative-based commercial chiral columns and chemical structure of the chiral selectors.

Commercial Name	Chiral Selector	Chemical Structure of the Chiral Selector
Lux^®^ Amylose-1, Chiralpak^®^ AD-H, Chiralpak^®^ AD-3R, Chiralpak^®^ AD, Lux^®^ Amylose-1, Chiralpak^®^ AD-H, YMC CHIRAL ART Amylose-C, Trefoil AMY1	coated amylose *tris*-(3,5-dimethylphenyl carbamate)	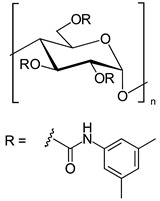
Lux^®^ i-amylose-1, Chiralpak^®^ IA, Chiralpak^®^ IG, Chiralpak^®^ IA-3	immobilized amylose *tris*-(3,5-dimethylphenyl carbamate)	
Lux^®^ amylose-2, Chiralpak^®^ AY-3R	coated amylose *tris*-(2-methyl-5-chlorophenyl carbamate)	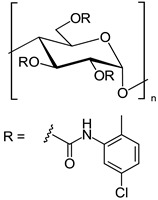
Chiralpak^®^ ID	immobilized amylose *tris*-(3,5-chlorophenyl carbamate)	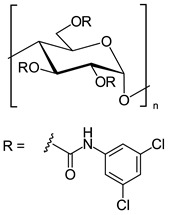
Chiralpak^®^ IE, Chiralpak^®^ ID-U	immobilized amylose *tris*-(3,5-dichlorophenyl carbamate)	
Chiralpak^®^ AZ-3R	coated amylose *tris*-(3-chloro-4-methylphenyl carbamate)	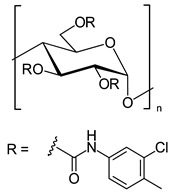
Chiralpak^®^ IF	immobilized amylose *tris*-(3-chloro-4-methylphenyl carbamate)	
Chiralpak^®^ IG, Lux^®^ i-Amylose-3, Chiralpak^®^ IG, Chiralpak^®^ IG-3, Chiralpak^®^ IG-U	immobilized amylose *tris*-(3-chloro-5-methylphenyl carbamate)	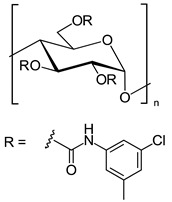
Chiralpak^®^ AS-3R	coated amylose *tris*-((*S*)-*α*-methylbenzyl carbamate)	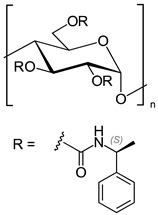

## Supplementary Materials

The following are available online, Figures S1–S40: Figure S1. Chemical structure of the cellulose 3,5-dichlorophenyl carbamatealkoxysilane hybrid CSPs; Figure S2. Chemical structure of the dialdehyde cellulose-derived CSP immobilized onto aminopropyl-modified silica gel by a Schiff´s base reaction; Figure S3. Chemical structures of the acrylate-type cellulose 3,5-dimethylphenyl carbamate-based CS (1) and the CSP obtained after thiol-ene addition (2); Figure S4. Chemical structure of pesticide analytes; Figure S5. Chemical structures of the chiral sulfoxides; Figure S6. Chemical structures of linolenic acids; Figure S7. Chemical structures of eight imidazole-type antifungal drug enantiomers; Figure S8. Chemical structures of six β-adrenergic blockers; Figure S9. Chemical structures of chiral imidazoline derivatives; Figure S10. Chemical structures of the medetomidine enantiomers; Figure S11. Chemical structures of clausenamidone and neoclausenamidone; Figure S12. Chemical structure of 6-(4-aminophenyl)-5-methyl-4,5-dihydro-3(2*H*)-pyridazinone; Figure S13. Chemical structures of ivabradine and its derivatives; Figure S14. Chemical structure of chiral guaifenesin; Figure S15. Chemical structures of amylose derivative-based CSs; Figure S16. Chemical structure of Corey lactone diol enantiomers; Figure S17. Chemical structure of the chiral drugs and 1-aryl-5-aryl-pyrrolidin-2-one derivatives; Figure S18. Chemical structure of 4C-substituted pyrrolidin-2-one derivatives; Figure S19. The chemical structures of chiral pesticides; Figure S20. The chemical structure of lumefantrine; Figure S21. The chemical structure of 5-[1-(4-nitrobenzylsulfonyloxy)-ethyl]-5-(pyridine-2-yl)-[1,3,4]-thiadiazole; Figure S22. Structures of α-cypermethrin and tetramethrin; Figure S23. Chemical structure of carvedilol; Figure S24. The chemical structures of amphetamine, pseudoephedrine, and related compounds; Figure S25. Chemical structures of the chiral amino compounds; Figure S26. Chemical structures of nine profens studied in fish tissue; Figure S27. Chemical structure of chitosan 3,6-bis-(4-methylphenyl carbamate)-2-(isobutyrylamide)-based CSs; Figure S28. Chemical structure of chitosan 3,6-bis(phenylcarbamate)-2-(cyclobutylformamide)-based CSs; Figure S29. Chemical structure of the chitosan derivatives-based CSs; Figure S30. Chemical structure of the stilbene diamido-bridged bis-(β-cyclodextrin) bonded silica gel-based CSP; Figure S31. Chemical structure of an ethylenediamine dicarboxyethyl diacetamidobridged bis-(β-cyclodextrin) bound to silica gel as CSP; Figure S32. Structures of the chiral analytes used in the study of Zhao et al.; Figure S33. Chemical structure of chiral analytes used in the study of Ke et al.; Figure S34. Chemical structure of benzylureido-β-cyclodextrin bound to silica gel as CSP; Figure S35. Chemical structure of phenylmercapturic and benzylmercapturic acid; Figure S36. Chemical structure of rasagiline; Figure S37. The chemical structure of lansoprazole and rabeprazole; Figure S38. Chemical structure of milnacipran; Figure S39. Chemical structure of the chiral analytes used in the study of Casado et al.; Figure S40. Chemical structures of sertraline isomers.

## References

[1] Berthod A. (2006). Chiral Recognition Mechanisms. Anal. Chem..

[2] Lämmerhofer M. (2010). Chiral recognition by enantioselective liquid chromatography: Mechanisms and modern chiral stationary phases. J. Chromatogr. A.

[3] Fanali C., D’Orazio G., Gentili A., Fanali S. (2019). Analysis of Enantiomers in Products of Food Interest. Molecules.

[4] D’Orazio G. (2020). Chiral analysis by nano-liquid chromatography. TrAC Trends Anal. Chem..

[5] Singh M., Sethi S., Bhushan R. (2020). Liquid chromatographic methods for separation, determination, and bioassay of enantiomers of etodolac: A review. J. Sep. Sci..

[6] Carrão D.B., Perovani I.S., de Albuquerque N.C.P., de Oliveira A.R.M. (2019). Enantioseparation Of Pesticides: A Critical Review. TrAC Trends Anal. Chem..

[7] Zhao P., Dong X., Chen X., Guo X., Zhao L. (2019). Stereoselective Analysis of Chiral Pyrethroid Insecticides Tetramethrin and α-Cypermethrin in Fruits, Vegetables, and Cereals. J. Agric. Food Chem..

[8] Chankvetadze B. (2019). Polysaccharide-Based Chiral Stationary Phases for Enantioseparations by High-Performance Liquid Chromatography: An Overview. Methods in Molecular Biology.

[9] Ianni F., Saluti G., Galarini R., Fiorito S., Sardella R., Natalini B. (2019). Enantioselective high-performance liquid chromatography analysis of oxygenated polyunsaturated fatty acids. Free. Radic. Biol. Med..

[10] Scriba G.K.E. (2019). Recognition Mechanisms of Chiral Selectors: An Overview. Methods in Molecular Biology.

[11] Teixeira J., Tiritan M.E., Pinto M.M., Fernandes C. (2019). Chiral stationary phases for liquid chromatography: Recent developments. Molecules.

[12] Hettegger H., Lindner W., Rosenau T. (2020). Derivatized polysaccharides on silica and hybridized with silica in chromatography and separation—A mini review. Recent Trends Carbohydr. Chem..

[13] Chankvetadze B. (2020). Recent trends in preparation, investigation and application of polysaccharide-based chiral stationary phases for separation of enantiomers in high-performance liquid chromatography. TrAC Trends Anal. Chem..

[14] Felletti S., Ismail O.H., De Luca C., Costa V., Gasparrini F., Pasti L., Marchetti N., Cavazzini A., Catani M. (2019). Recent Achievements and Future Challenges in Supercritical Fluid Chromatography for the Enantioselective Separation of Chiral Pharmaceuticals. Chromatographia.

[15] Yin C., Zhang J., Chang L., Zhang M., Yang T., Zhang X., Zhang J. (2019). Regioselectively substituted cellulose mixed esters synthesized by two-steps route to understand chiral recognition mechanism and fabricate high-performance chiral stationary phases. Anal. Chim. Acta.

[16] Felix G. (2001). Regioselectively modified polysaccharide derivatives as chiral stationary phases in high-performance liquid chromatography. J. Chromatogr. A.

[17] Acemoglu M., Hernandez I., Mak C.P. (1998). Synthesis of regioselectively substituted cellulose derivatives and applications in chiral chromatography. Chirality.

[18] Katoh Y., Tsujimoto Y., Yamamoto C., Ikai T., Kamigaito M., Okamoto Y. (2011). Chiral recognition ability of cellulose derivatives bearing pyridyl and bipyridyl residues as chiral stationary phases for high-performance liquid chromatography. Polym. J..

[19] Yu X., Wang Y., Yang Q., Zhang Z., Ren Q., Bao Z., Yang Y. (2020). De novo synthesis of microspherical cellulose 3,5-dichlorophenylcarbamates: An organic-inorganic hybrid chiral stationary phase for enantioseparation. Sep. Purif. Technol..

[20] Qian H., Shen X., Huang H., Zhang Y., Zhang M., Wang H., Wang Z., Wang Z. (2020). Helical poly(phenyl isocyanide)s grafted selectively on C-6 of cellulose for improved chiral recognition ability. Carbohydr. Polym..

[21] Gao J., Quan K., Li H., Li Z., Zhao L., Qiu H. (2020). Preparation and evaluation of biselector bonded-type multifunctional chiral stationary phase based on dialdehyde cellulose and 6-monodeoxy-6-monoamino-β-cyclodextrine derivatives. Chirality.

[22] Gao J., Chen L., Wu Q., Li H., Dong S., Qin P., Yang F., Zhao L. (2019). Preparation and chromatographic performance of a multifunctional immobilized chiral stationary phase based on dialdehyde microcrystalline cellulose derivatives. Chirality.

[23] Siller M., Amer H., Bacher M., Roggenstein W., Rosenau T., Potthast A. (2015). Effects of periodate oxidation on cellulose polymorphs. Cellulose.

[24] Gao J., Luo G., Li Z., Li H., Zhao L., Qiu H. (2020). A new strategy for the preparation of mixed-mode chromatographic stationary phases based on modified dialdehyde cellulose. J. Chromatogr. A.

[25] Plappert S.F., Quraishi S., Pircher N., Mikkonen K., Veigel S., Klinger K.M., Potthast A., Rosenau T., Liebner F.W. (2018). Transparent, Flexible, and Strong 2,3-Dialdehyde Cellulose Films with High Oxygen Barrier Properties. Biomacromolecules.

[26] Yin C., Chen W., Zhang J., Zhang M., Zhang J. (2019). A facile and efficient method to fabricate high-resolution immobilized cellulose-based chiral stationary phases via thiol-ene click chemistry. Sep. Purif. Technol..

[27] Li L., Wang H., Shuang Y., Li L. (2019). The preparation of a new 3, 5-dichlorophenylcarbamated cellulose-bonded stationary phase and its application for the enantioseparation and determination of chiral fungicides by LC-MS/MS. Talanta.

[28] Echevarría R.N., Keunchkarian S., Villarroel-Rocha J., Sapag K., Reta M. (2019). Organic monolithic capillary columns coated with cellulose tris(3,5-dimethylphenyl carbamate) for enantioseparations by capillary HPLC. Microchem. J..

[29] Carradori S., Secci D., Guglielmi P., Pierini M., Cirilli R. (2020). High-performance liquid chromatography enantioseparation of chiral 2-(benzylsulfinyl)benzamide derivatives on cellulose tris(3,5-dichlorophenylcarbamate) chiral stationary phase. J. Chromatogr. A.

[30] Ianni F., Blasi F., Giusepponi D., Coletti A., Galli F., Chankvetadze B., Galarini R., Sardella R. (2020). Liquid chromatography separation of α- and γ-linolenic acid positional isomers with a stationary phase based on covalently immobilized cellulose tris(3,5-dichlorophenylcarbamate). J. Chromatogr. A.

[31] Zhang J., Sun J., Liu Y., Yu J., Guo X. (2019). Immobilized Cellulose-Based Chiralpak IC Chiral Stationary Phase for Enantiosep-aration of Eight Imidazole Antifungal Drugs in Normal-Phase, Polar Organic Phase and Reversed-Phase Conditions Using High-Performance Liquid Chromatography. Chromatographia.

[32] Li M., Jiang Z., Di X., Song Y. (2020). Enantiomeric separation of six beta-adrenergic blockers on Chiralpak IB column and iden-tification of chiral recognition mechanisms by molecular docking technique. Biomed. Chromatogr..

[33] Cerra B., Macchiarulo A., Carotti A., Camaioni E., Varfaj I., Sardella R., Gioiello A. (2020). Enantioselective HPLC Analysis to Assist the Chemical Exploration of Chiral Imidazolines. Molecules.

[34] Karakka Kal A.K., Nalakath J., Kunhamu Karatt T., Perwad Z., Mathew B., Subhahar M. (2020). Development and validation of a chiral LC-MS method for the enantiomeric resolution of (+) and (−)-medetomidine in equine plasma by using polysaccharide-based chiral stationary phases. Chirality.

[35] Tanács D., Orosz T., Szakonyi Z., Le T.M., Fülöp F., Lindner W., Ilisz I., Péter A. (2020). High-performance liquid chromatographic enantioseparation of isopulegol-based ß-amino lactone and ß-amino amide analogs on polysaccharide-based chiral stationary phases focusing on the change of the enantiomer elution order. J. Chromatogr. A.

[36] Luo X., Fang C., Mi J., Xu J., Lin H. (2019). Enantiomeric resolution, thermodynamic parameters, and modeling of clausenamidone and neoclausenamidone on polysaccharide-based chiral stationary phases. Chirality.

[37] Cheng L., Cai J., Fu Q., Ke Y. (2019). Efficient preparative separation of 6-(4-aminophenyl)-5-methyl-4,5-dihydro-3 (2H)-pyridazinone enantiomers on polysaccharide-based stationary phases in polar organic solvent chromatography and super-critical fluid chromatography. J. Sep. Sci..

[38] Orosz T., Bajtai A., Minh Le T., Tanács D., Szakonyi Z., Fülöp F., Péter A., Ilisz I. (2019). Chiral high-performance liquid and supercritical fluid chromatographic enantioseparations of limonene-based bicyclic aminoalcohols and aminodiols on polysaccharide-based chiral stationary phases. Biomed. Chromatogr..

[39] Ferencz E., Kovács B., Boda F., Foroughbakhshfasaei M., Kelemen É.K., Tóth G., Szabó Z.-I. (2020). Simultaneous determination of chiral and achiral impurities of ivabradine on a cellulose tris(3-chloro-4-methylphenylcarbamate) chiral column using polar organic mode. J. Pharm. Biomed. Anal..

[40] Tantawy M.A., Yehia A.M., Aboul-Enein H.Y. (2019). Simultaneous determination of guaifenesin enantiomers and ambroxol HCl using 50-mm chiral column for a negligible environmental impact. Chirality.

[41] Cutillas V., García-Valverde M., del Mar Gómez-Ramos M., Díaz-Galiano F.J., Ferrer C., Fernández-Alba A.R. (2020). Supercritical fluid chromatography separation of chiral pesticides: Unique capabilities to study cyhalothrin and metalaxyl as examples. J. Chromatogr. A.

[42] Douša M. (2019). Enantioseparation of *N*-acetyl-DL-cysteine as *o*-phthaldialdehyde derivatives obtained with various primary ali-phatic amine additives on polysaccharide-based chiral stationary phases. J. Pharm. Biomed. Anal..

[43] Dai X., Bi W., Sun M., Wang F., Shen J., Okamoto Y. (2019). Chiral recognition ability of amylose derivatives bearing regioselectively different carbamate pendants at 2,3- and 6-positions. Carbohydr. Polym..

[44] Ryoki A., Kimura Y., Kitamura S., Maeda K., Terao K. (2019). Does local chain conformation affect the chiral recognition ability of an amylose derivative? Comparison between linear and cyclic amylose tris(3,5-dimethylphenylcarbamate). J. Chromatogr. A.

[45] Maisuradze M., Sheklashvili G., Chokheli A., Matarashvili I., Gogatishvili T., Farkas T., Chankvetadze B. (2019). Chromatographic and thermodynamic comparison of amylose tris(3-chloro-5-methylphenylcarbamate) coated or covalently immobilized on silica in high-performance liquid chromatographic separation of the enantiomers of select chiral weak acids. J. Chromatogr. A.

[46] Wang H., Wang Q., Wu Y., Cheng L., Zhu L., Zhu J., Ke Y. (2019). HPLC and SFC enantioseparation of (±)-Corey lactone diol: Impact of the amylose tris-(3, 5-dimethylphenylcarbamate) coating amount on chiral preparation. Chirality.

[47] Dascalu A.-E., Ghinet A., Chankvetadze B., Lipka E. (2020). Comparison of dimethylated and methylchlorinated amylose stationary phases, coated and covalently immobilized on silica, for the separation of some chiral compounds in supercritical fluid chromatography. J. Chromatogr. A.

[48] Upmanis T., Kažoka H., Orlova N., Vorona M. (2020). Separation of 4C-Substituted Pyrrolidin-2-One Derivatives on Polysaccha-ride-Based Coated Chiral Stationary Phases. Chromatographia.

[49] Zhao P., Li S., Chen X., Guo X., Zhao L. (2019). Simultaneous enantiomeric analysis of six chiral pesticides in functional foods using magnetic solid-phase extraction based on carbon nanospheres as adsorbent and chiral liquid chromatography coupled with tandem mass spectrometry. J. Pharm. Biomed. Anal..

[50] Kadkhodaei K., Kadisch M., Schmid M.G.J.C. (2020). Successful use of a novel lux® i-Amylose-1 chiral column for enantioseparation of “legal highs” by HPLC. J. Chirality.

[51] Kim T., Bao C., Hausmann M., Siqueira G., Zimmermann T., Kim W.S. (2019). 3D Printed Disposable Wireless Ion Sensors with Biocompatible Cellulose Composites. Adv. Electron. Mater..

[52] Rane V.P., Ahirrao V.K., Patil K.R., Jadhav R., Ingle R.G., More K.B., Yeole R.D. (2019). Enantiomeric Separation and Thermodynamic investigation of (R)-5-[1-(4-Nitrobenzylsulfonyloxy)-ethyl]-5-(pyridine-2-yl)-[1,3,4]-thiadiazole, a Key Intermediate of Nafithromycin. Anal. Chem. Lett..

[53] Merino M.E.D., Echevarría R.N., Lubomirsky E., Padró J.M., Castells C.B. (2019). Enantioseparation of the racemates of a number of pesticides on a silica-based column with immobilized amylose tris(3-chloro-5-methylphenylcarbamate). Microchem. J..

[54] D’Orazio G., Fanali C., Fanali S., Gentili A., Chankvetadze B. (2019). Comparative study on enantiomer resolving ability of amylose tris(3-chloro-5-methylphenylcarbamate) covalently immobilized onto silica in nano-liquid chromatography and capillary electrochromatography. J. Chromatogr. A.

[55] Panella C., Ferretti R., Casulli A., Cirilli R. (2019). Temperature and eluent composition effects on enantiomer separation of carvedilol by high-performance liquid chromatography on immobilized amylose-based chiral stationary phases. J. Pharm. Anal..

[56] Karakka Kal A.K., Karatt T.K., Sayed R., Philip M., Meissir S., Nalakath J. (2019). Separation of ephedrine and pseudoephedrine enantiomers using a polysaccharide-based chiral column: A normal phase liquid chromatography–high-resolution mass spectrometry approach. Chirality.

[57] Ibrahim D., Ghanem A. (2019). On the Enantioselective HPLC Separation Ability of Sub-2 µm Columns: Chiralpak® IG-U and ID-U. Molecues.

[58] Bajtai A., Lajkó G., Németi G., Szatmári I., Fülöp F., Péter A., Ilisz I. (2019). High-performance liquid chromatographic and subcritical fluid chromatographic separation of α-arylated ß-carboline, *N*-alkylated tetrahydroisoquinolines and their bioisosteres on polysaccharide-based chiral stationary phases. J. Sep. Sci..

[59] Petrovaj J., Kudličková Z., Budovská M., Salayová A., Baláž M., Lindner W., Gondová T. (2019). Liquid chromatographic chiral recognition of phytoalexins on immobilized polysaccharides chiral stationary phases. Unusual temperature behavior. J. Chromatogr. A.

[60] Kozlov O., Kalíková K., Gondová T., Budovská M., Salayová A., Tesařová E. (2019). Fast enantioseparation of indole phytoalexins in additive free supercritical fluid chromatography. J. Chromatogr. A.

[61] Li M., Liang X., Guo X., Di X., Jiang Z. (2020). Enantiomeric separation and enantioselective determination of some representative non-steroidal anti-inflammatory drug enantiomers in fish tissues by using chiral liquid chromatography coupled with tandem mass spectrometry. Microchem. J..

[62] Zhang G.-H., Xi J.-B., Chen W., Bai Z.-W. (2020). Comparison in enantioseparation performance of chiral stationary phases prepared from chitosans of different sources and molecular weights. J. Chromatogr. A.

[63] Fu L.-L., Wang X.-C., Fu K.-Q., Xi J.-B., Chen W., Tang S., Bai Z.-W. (2019). Dependence of enantioseparation performance on structure of chiral selectors derived from *N*-cycloalkylcarbonyl chitosan. React. Funct. Polym..

[64] Zhang J., Zhang G.-H., Wang X.-C., Bai Z.-W., Chen W. (2019). Synthesis and evaluation of novel chiral stationary phases based on *N*-cyclobutylcarbonyl chitosan derivatives. Microchem. J..

[65] Zhang G.-H., Liang S., Tang S., Chen W., Bai Z.-W. (2019). Performance evaluation of enantioseparation materials based on chitosan isobutylurea derivatives. Anal. Methods.

[66] Yang X., Niu X., Mo Z., Guo R., Liu N., Zhao P., Liu Z. (2019). Perylene-functionalized graphene sheets modified with chitosan for voltammetric discrimination of tryptophan enantiomers. Microchim. Acta.

[67] Xiao X., Li Z., Liu Y., Jia L. (2019). Preparation of chitosan-based molecularly imprinted material for enantioseparation of racemic mandelic acid in aqueous medium by solid phase extraction. J. Sep. Sci..

[68] Shuang Y., Liao Y., Wang H., Wang Y., Li L. (2020). Preparation and evaluation of a triazole-bridged bis(β-cyclodextrin)–bonded chiral stationary phase for HPLC. Chirality.

[69] Shuang Y., Zhang T., Li L. (2020). Preparation of a stilbene diamido-bridged bis(β-cyclodextrin)-bonded chiral stationary phase for enantioseparations of drugs and pesticides by high performance liquid chromatography. J. Chromatogr. A.

[70] Shuang Y., Liao Y., Zhang T., Li L. (2020). Preparation and evaluation of an ethylenediamine dicarboxyethyl diamido-bridged bis(β-cyclodextrin)-bonded chiral stationary phase for high performance liquid chromatography. J. Chromatogr. A.

[71] Ji J., Wu W., Liang W., Cheng G., Matsushita R., Yan Z., Wei X., Rao M., Yuan D.-Q., Fukuhara G. (2019). An Ultimate Stereocontrol in Supramolecular Photochirogenesis: Photocyclodimerization of 2-Anthracenecarboxylate Mediated by Sulfur-Linked β-Cyclodextrin Dimers. J. Am. Chem. Soc..

[72] Wei X., Wu W., Matsushita R., Yan Z., Zhou D., Chruma J.J., Nishijima M., Fukuhara G., Mori T., Inoue Y. (2018). Supramolecular Photochirogenesis Driven by Higher-Order Complexation: Enantiodifferentiating Photocyclodimerization of 2-Anthracenecarboxylate to Slipped Cyclodimers via a 2:2 Complex with β-Cyclodextrin. J. Am. Chem. Soc..

[73] Dai L., Wu W., Liang W., Chen W.-T., Yu X., Ji J., Xiao C., Yang C. (2018). Enhanced chiral recognition by γ-cyclodextrin–cucurbit- [6]uril-cowheeled [4] pseudorotaxanes. Chem. Commun..

[74] Bálint M., Darcsi A., Benkovics G., Varga E., Malanga M., Béni S. (2019). Synthesis of the chiral selector heptakis(6-O-methyl)-β-cyclodextrin by phase-transfer catalysis and hydrazine-mediated transfer-hydrogenation. Electrophoresis.

[75] Sun J., Ma S., Liu B., Yu J., Guo X. (2019). A fully derivatized 4-chlorophenylcarbamate-β-cyclodextrin bonded chiral stationary phase for enhanced enantioseparation in HPLC. Talanta.

[76] Zhao Y., Wang J., Liu Y., Jiang Z., Song Y., Guo X. (2020). Enantioseparation using carboxymethyl-6-(4-methoxybenzylamino)-β-cyclodextrin as a chiral selector by capillary electrophoresis and molecular modeling study of the recognition mechanism. New J. Chem..

[77] Ren X., Luo Q., Zhou D., Zhang K., Gao D., Fu Q., Liu J., Xia Z., Wang L. (2020). Thermoresponsive chiral stationary phase functionalized with the copolymer of β-cyclodextrin and *N*-isopropylacrylamide for high performance liquid chromatography. J. Chromatogr. A.

[78] Ke J., Zhang Y., Zhang X., Liu Y., Ji Y., Chen J. (2020). Novel chiral composite membrane prepared *via* the interfacial polymer-ization of diethylamino-beta-cyclodextrin for the enantioseparation of chiral drugs. J. Membr. Sci..

[79] Li L., Wang H., Jin Y., Shuang Y., Li L. (2019). Preparation of a new benzylureido-β-cyclodextrin-based column and its application for the determination of phenylmercapturic acid and benzylmercapturic acid enantiomers in human urine by LC/MS/MS. Anal. Bioanal. Chem..

[80] Wu Q., Gao J., Chen L., Dong S., Li H., Qiu H., Zhao L. (2019). Graphene quantum dots functionalized β-cyclodextrin and cellulose chiral stationary phases with enhanced enantioseparation performance. J. Chromatogr. A.

[81] Naghdi E., Fakhari A.R., Baca M., De Malsche W. (2020). Simultaneous enantioseparation of nonsteroidal anti-inflammatory drugs by a one-dimensional liquid chromatography technique using a dynamically coated chiral porous silicon pillar array column. J. Chromatogr. A.

[82] Jiang Z., Qu J., Tian X., Huo X., Zhang J., Guo X., Fang L. (2019). Sol-gel technique for the preparation of β-cyclodextrin gold nanoparticles as chiral stationary phase in open-tubular capillary electrochromatography. J. Sep. Sci..

[83] Zhou L., Lun J., Liu Y., Jiang Z., Di X., Guo X. (2019). In situ immobilization of sulfated-β-cyclodextrin as stationary phase for capillary electrochromatography enantioseparation. Talanta.

[84] Wang T., Cheng Y., Zhang Y., Zha J., Ye J., Chu Q., Cheng G. (2020). β-cyclodextrin modified quantum dots as pseudo-stationary phase for direct enantioseparation based on capillary electrophoresis with laser-induced fluorescence detection. Talanta.

[85] Chen F., Fan Z., Zhu Y., Sun H., Yu J., Jiang N., Zhao S., Lai G., Yu A., Lin C.-T. (2020). β-Cyclodextrin-Immobilized Ni/Graphene Electrode for Electrochemical Enantiorecognition of Phenylalanine. Materials.

[86] Sun X., Tao Y., Du Y., Ding W., Chen C., Ma X. (2019). Metal organic framework HKUST-1 modified with carboxymethyl-β-cyclodextrin for use in improved open tubular capillary electrochromatographic enantioseparation of five basic drugs. Microchim. Acta.

[87] Deng M., Li S., Cai L., Guo X. (2019). Preparation of a hydroxypropyl-β-cyclodextrin functionalized monolithic column by one-pot sequential reaction and its application for capillary electrochromatographic enantiomer separation. J. Chromatogr. A.

[88] Yang X., Sun X., Feng Z., Du Y., Chen J., Ma X., Li X. (2019). Open-tubular capillary electrochromatography with β-cyclodextrin-functionalized magnetic nanoparticles as stationary phase for enantioseparation of dansylated amino acids. Microchim. Acta.

[89] Sun X., Guo J., Yu T., Du Y., Feng Z., Zhao S., Huang Z., Liu J. (2019). A novel coating method for CE capillary using carbox-ymethyl-β-cyclodextrin-modified magnetic microparticles as stationary for electrochromatography enantioseparation. Anal. Bioanal. Chem..

[90] Sun X., Du Y., Zhao S., Huang Z., Feng Z. (2019). Enantioseparation of propranolol, amlodipine and metoprolol by electro-chromatography using an open tubular capillary modified with β-cyclodextrin and poly(glycidyl methacrylate) nanoparticles. Microchim. Acta.

[91] Khatri S., Memon N., Khatri Z., Ahmed F. (2020). TLC-based enantiomeric separation of amino acids onto β-CD-incorporated glutaraldehyde-crosslinked PVA electrospun fiber stationary phase. Acta Chromatogr..

[92] Folprechtová D., Kalíková K., Kozlík P., Tesařová E. (2019). The degree of substitution affects the enantioselectivity of sulfobutylether-β-cyclodextrin chiral stationary phases. Electrophoresis.

[93] Szabó Z.-I., Ludmerczki R., Fiser B., Noszál B., Tóth G. (2019). Chiral separation of rasagiline using sulfobutylether-β-cyclodextrin: Capillary electrophoresis, NMR and molecular modeling study. Electrophoresis.

[94] Papp L.A., Hancu G., Gyéresi Á., Kelemen H., Szabó Z., Noszál B., Dubský P., Tóth G. (2019). Chiral separation of lansoprazole and rabeprazole by capillary electrophoresis using dual cyclodextrin systems. Electrophoresis.

[95] Pathak P., Coutinho E.C., Mohanraj K., Martis E., Jain V. (2020). Chromatographic and Computational Studies on the Chiral Recognition of Sulfated β-Cyclodextrin on Enantiomeric Separation of Milnacipran. Anal. Chem..

[96] Hägele J.S., Hubner E.M., Schmid M.G. (2019). Chiral separation of cathinone derivatives using β-cyclodextrin-assisted capillary electrophoresis–Comparison of four different β-cyclodextrin derivatives used as chiral selectors. Electrophoresis.

[97] Shi X., Zhou Y., Liu F., Mao J., Zhang Y., Shan T. (2019). Modeling of chiral gas chromatographic separation of alkyl and cycloalkyl 2-bromopropionates using cyclodextrin derivatives as stationary phases. J. Chromatogr. A.

[98] Casado N., Saz J.M., García M.Á., Marina M.L. (2020). Modeling-based optimization of the simultaneous enantiomeric separation of multicomponent mixtures of phenoxy acid herbicides using dual cyclodextrin systems by Capillary Electrophoresis. J. Chromatogr. A.

[99] Sun W., Wang C., Jin Y., Wang X., Zhao S., Luo M., Yan J., Tong S. (2019). Stereoselective separation of (1S,4S)-sertraline from medicinal reaction mixtures by countercurrent chromatography with hydroxypropyl-β-cyclodextrin as stereoselective selector. J. Sep. Sci..

[100] Yu T., Zhang J., Sun X., Du Y. (2019). Evaluation of cyclodextrin-micellar electrokinetic capillary chromatography with pyrrolidinium-based ionic liquid surfactant as a pseudostationary phase for chiral separation. Sep. Sci. Plus.

