# Supplementary material for: Polysaccharide- and β-Cyclodextrin-Based Chiral Selectors for Enantiomer Resolution: Recent Developments and Applications
Source: Molecules. 2021 Jul 16;26(14):4322. doi: 10.3390/molecules26144322 (PMC8307936; doi:10.3390/molecules26144322)
Supplement: Supplementary file 1 [file molecules-26-04322-s001.zip › molecules-1280332-supplementary.pdf]

SUPPORTING INFORMATION

Polysaccharide- and β-Cyclodextrin-based Chiral Selectors for Enantiomer Resolution: Recent Developments and Applications

Cuong Viet Bui[1,2], Thomas Rosenau[1,3], Hubert Hettegger[1,*]

[1] University of Natural Resources and Life Sciences, Vienna (BOKU), Department of Chemistry, Institute of Chemistry of Renewable Resources, Konrad-Lorenz-Straße 24, A-3430 Tulln, Austria

[2] University of Science and Technology, The University of Danang, Department of Food Technology, Faculty of Chemical Engineering, Danang City, 550000, Viet Nam

[3] Johan Gadolin Process Chemistry Centre, Åbo Akademi University, Porthansgatan 3, FI-20500 Åbo, Finland

* Corresponding Author: hubert.hettegger@boku.ac.at

Keywords

Amylose, Cellulose, Chiral Selector, Cyclodextrin, Enantiomer Separation, Polysaccharide

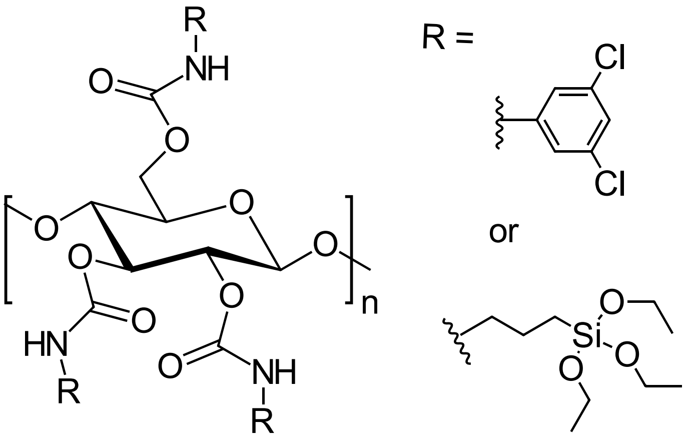

Figure S1. Chemical structure of the cellulose 3,5-dichlorophenyl carbamate-alkoxysilane hybrid CSPs (Yu et al., 2020)

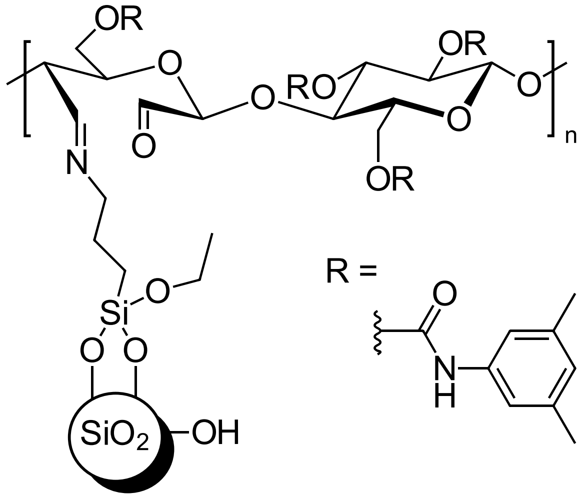

Figure S2. Chemical structure of the dialdehyde cellulose-derived CSP immobilized onto aminopropyl-modified silica gel by a Schiff´s base reaction (Gao et al., 2019). Note that the aldehyde groups are present in a complex equilibrium of hydrates, hemiacetals, and hemialdals (Amer et al., 2016)

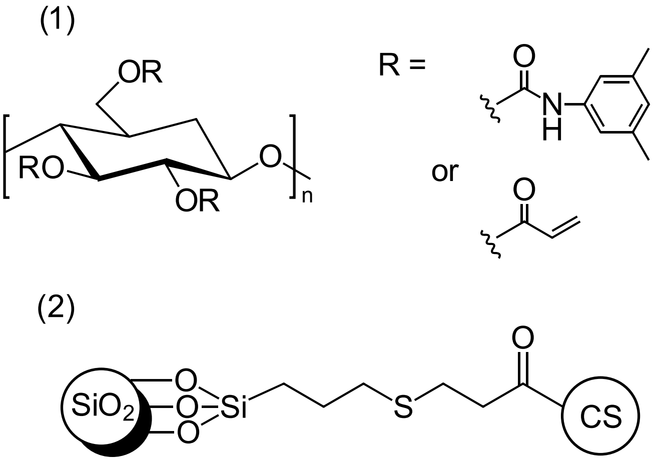

Figure S3. Chemical structures of the acrylate-type cellulose 3,5-dimethylphenyl carbamate-based CS (1) and the CSP obtained after thiol-ene addition (2) (Yin, Chen, Zhang, Zhang, & Zhang, 2019)

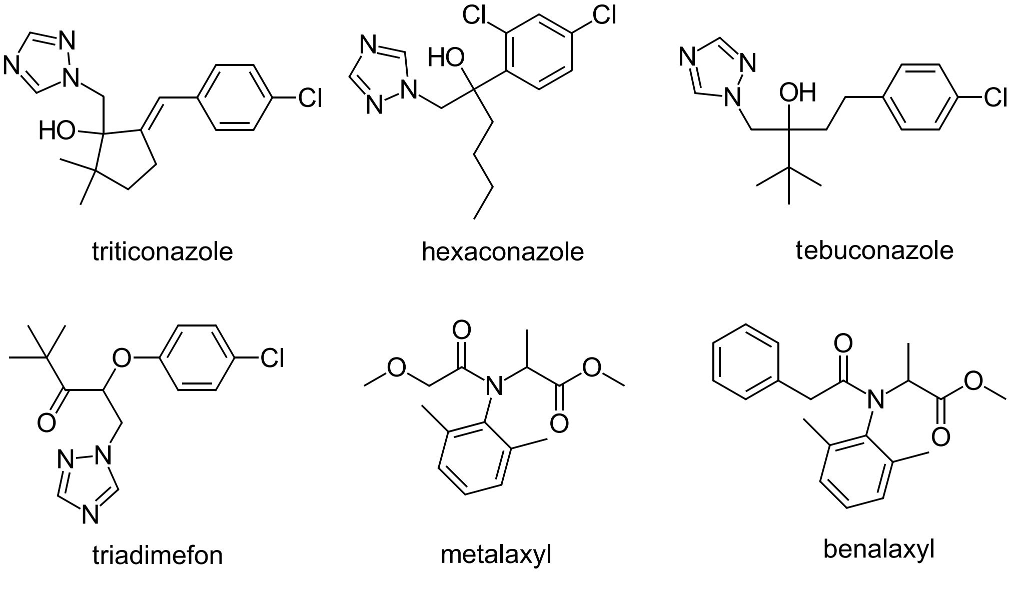

Figure S4. Chemical structure of pesticide analytes (L. Li, Wang, Shuang, & Li, 2019)

X =	R =
	Methyl
	Ethyl
$-NH_2$	*n*-Propyl
	n-Butyl
	n-Pentyl
	Methyl
	Ethyl
$-OCH_3$	*n*-Propyl
	n-Butyl
	n-Pentyl

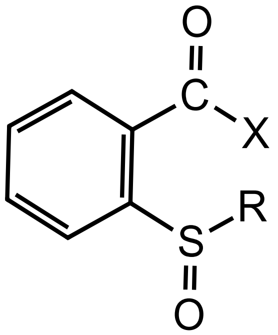

Figure S5. Chemical structures of the chiral sulfoxides (Carradori, Secci, Guglielmi, Pierini, & Cirilli, 2020)

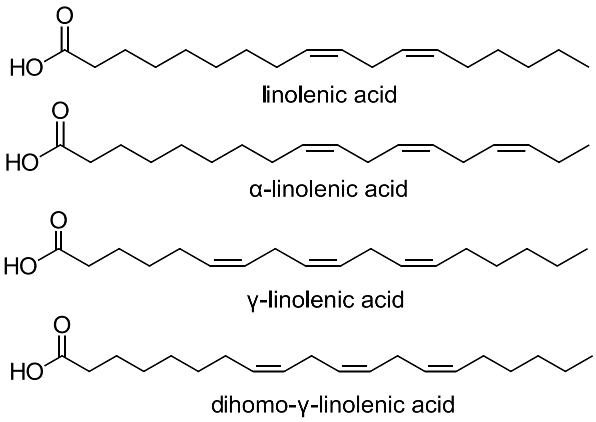

Figure S6. Chemical structures of linolenic acids (Ianni et al., 2020)

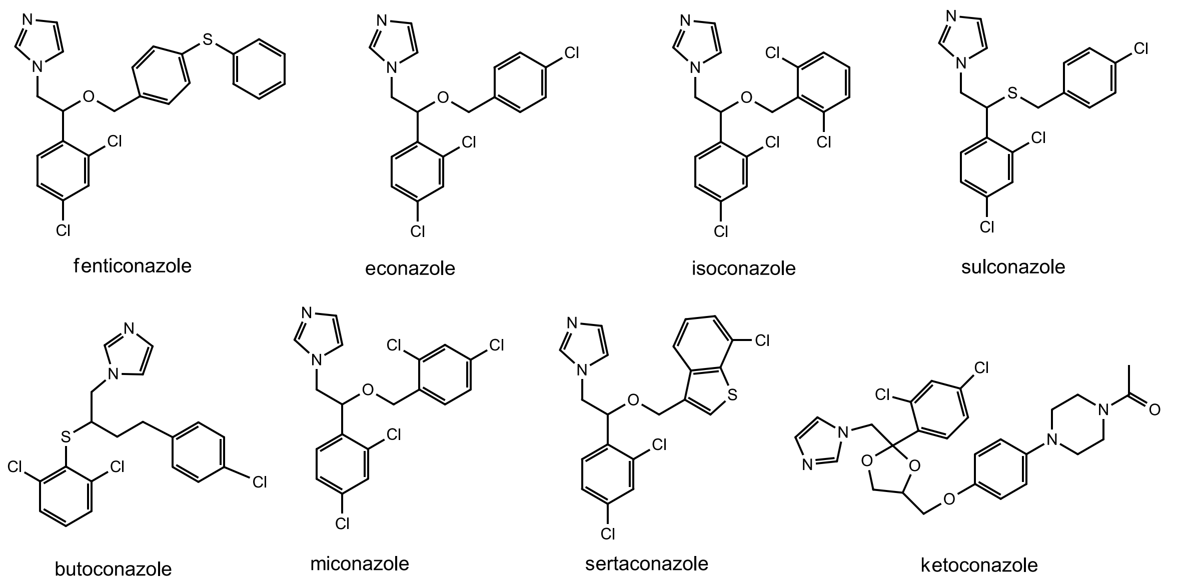

Figure S7. Chemical structures of eight imidazole-type antifungal drug enantiomers (J. Zhang, Sun, Liu, Yu, & Guo, 2019)

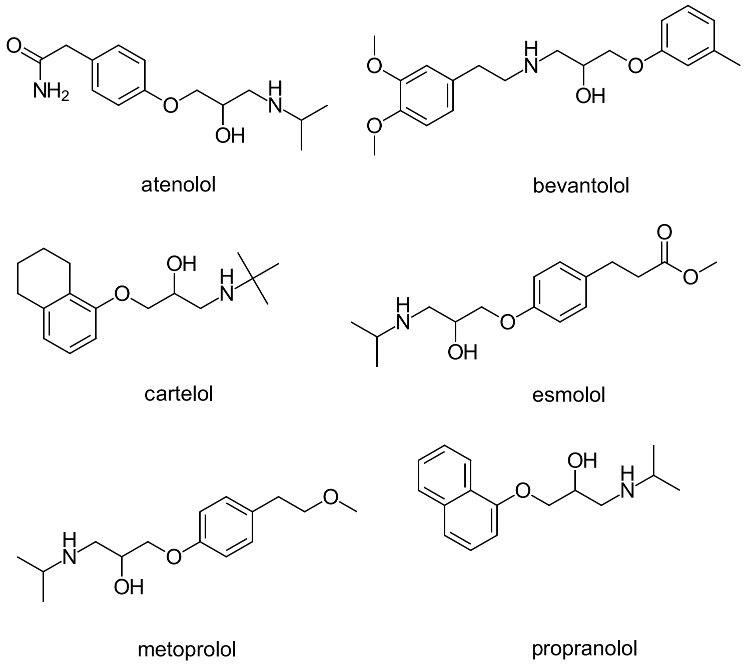

Figure S8. Chemical structures of six β-adrenergic blockers (M. Li, Jiang, Di, & Song, 2020)

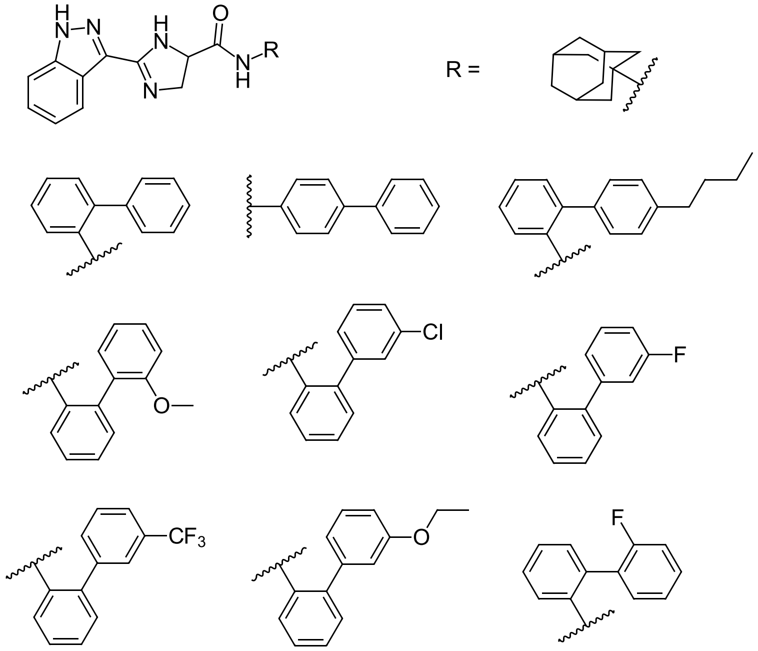

Figure S9. Chemical structures of chiral imidazoline derivatives (Cerra et al., 2020)

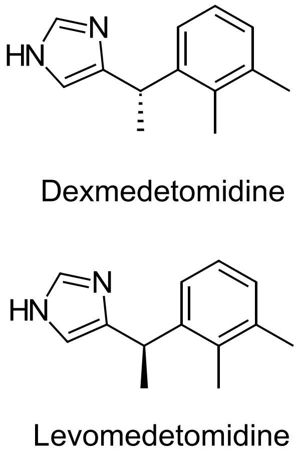

Figure S10. Chemical structures of the medetomidine enantiomers (Karakka Kal et al., 2020)

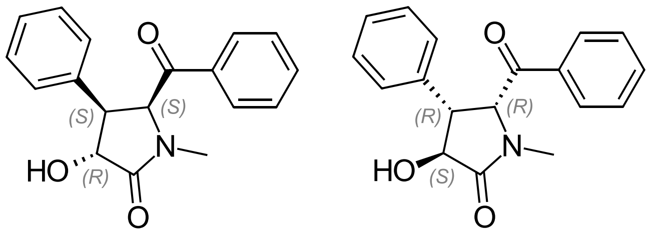

clausenamidone

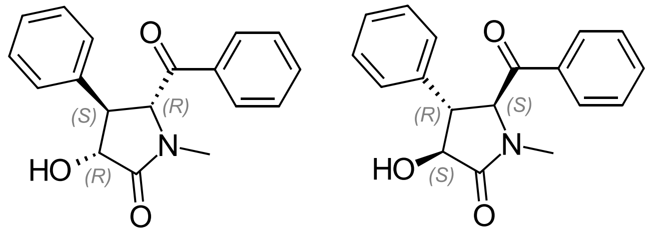

neoclausenamidone

Figure S11. Chemical structures of clausenamidone and neoclausenamidone (Luo, Fang, Mi, Xu, & Lin, 2019)

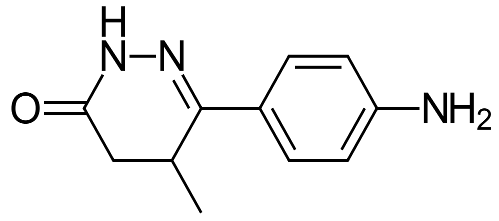

Figure S12. Chemical structure of 6-(4-aminophenyl)-5-methyl-4,5-dihydro-3(2H)-pyridazinone (Cheng, Cai, Fu, & Ke, 2019)

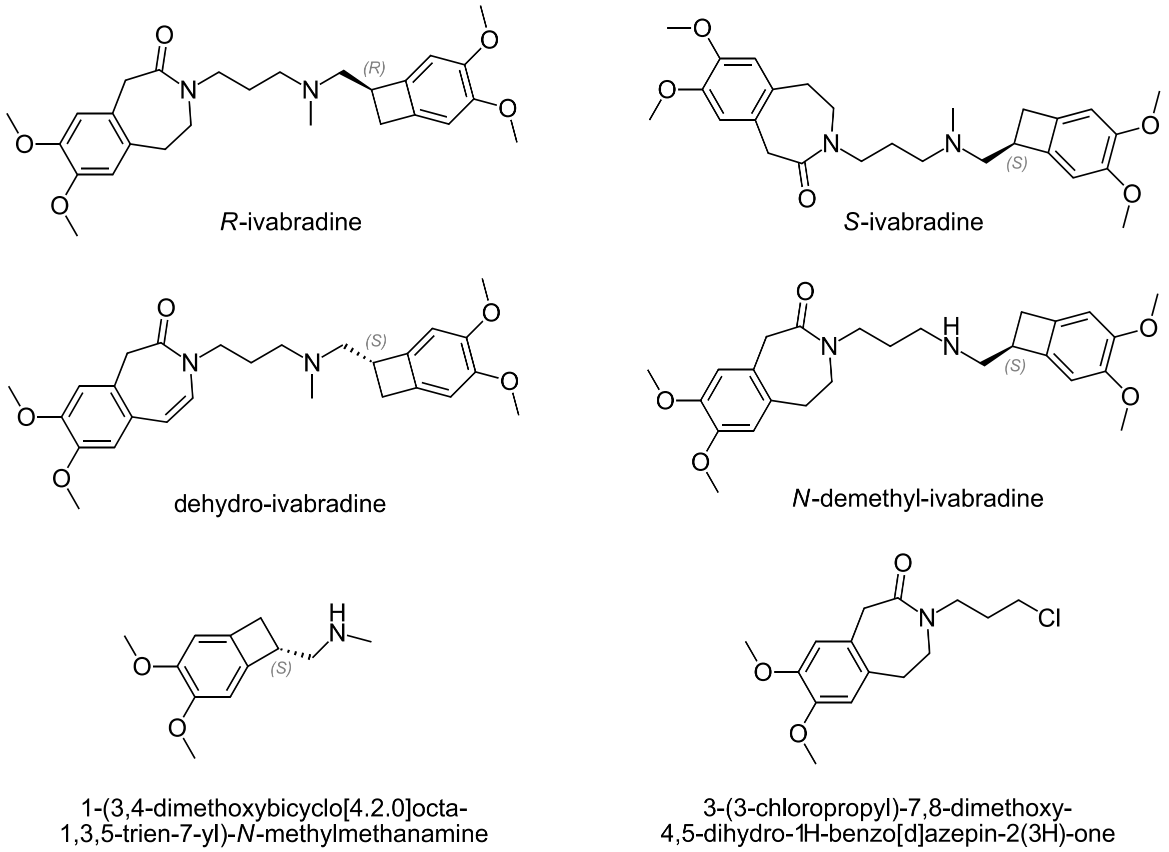

Figure S13. Chemical structures of ivabradine and its derivatives

(Ferencz et al., 2020)

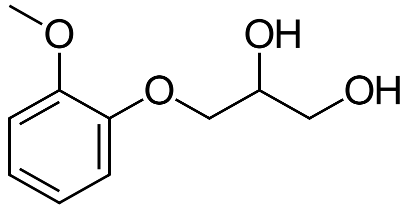

Figure S14. Chemical structure of chiral guaifenesin

(Tantawy, Yehia, & Aboul-Enein, 2019)

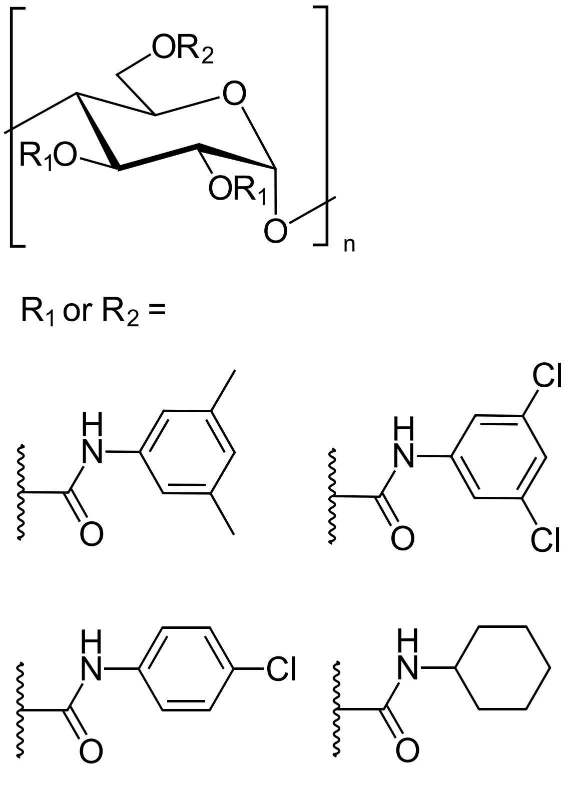

Figure S15. Chemical structures of amylose derivative-based CSs (Dai et al., 2019)

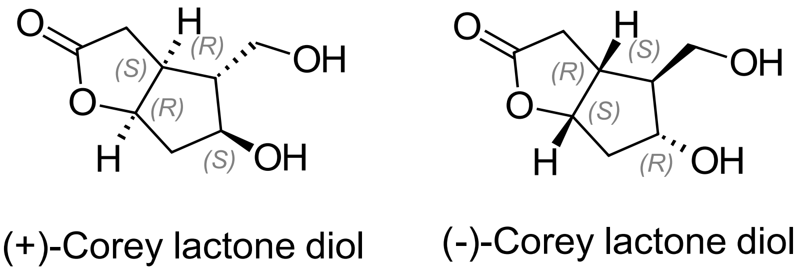

Figure S16. Chemical structure of Corey lactone diol enantiomers (Wang et al., 2019)

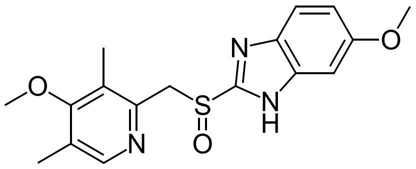

omeprazole

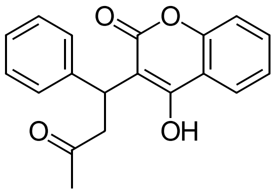

warfarin

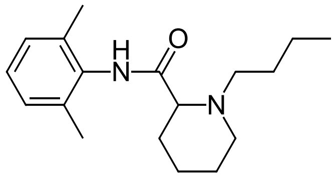

bupivacaine

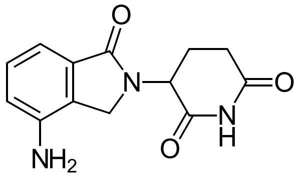

lenalidomide

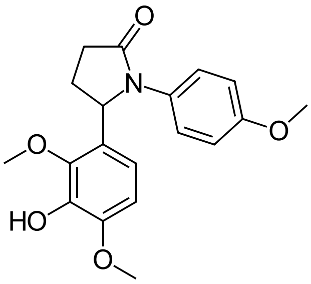

5-(3-hydroxy-2,4-dimethoxy-phenyl)-1-(4-methoxyphenyl)-pyrrolidin-2-one

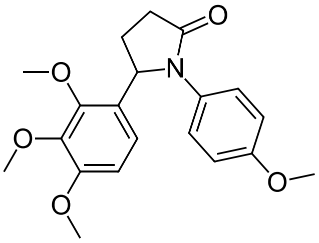

1-(4-methoxyphenyl)-5-(2,3,4-trimethoxyphenyl)-pyrrolidin-2-one

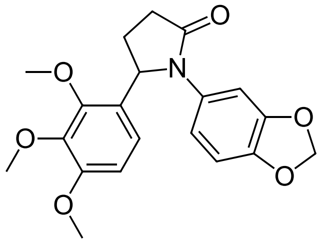

1-(benzo[d][1,3]dioxol-5-yl)-5-(2,3,4-trimethoxyphenyl)-pyrrolidin-2-one

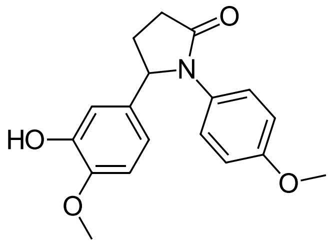

5-(3-hydroxy-4-methoxy-phenyl)-1-(4-methoxyphenyl)-pyrrolidin-2-one

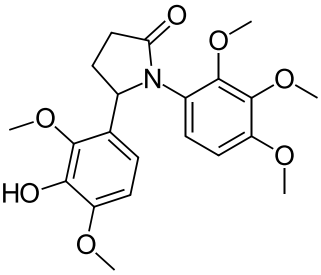

5-(3-hydroxy-2,4-dimethoxy-phenyl)-1-(2,3,4-trimethoxy-phenyl)pyrrolidin-2-one

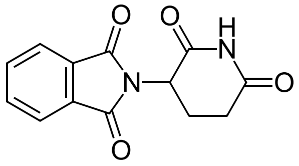

thalidomide

Figure S17. Chemical structure of the chiral drugs and 1-aryl-5-aryl-pyrrolidin-2-one derivatives (Dascalu, Ghinet, Chankvetadze, & Lipka, 2020)

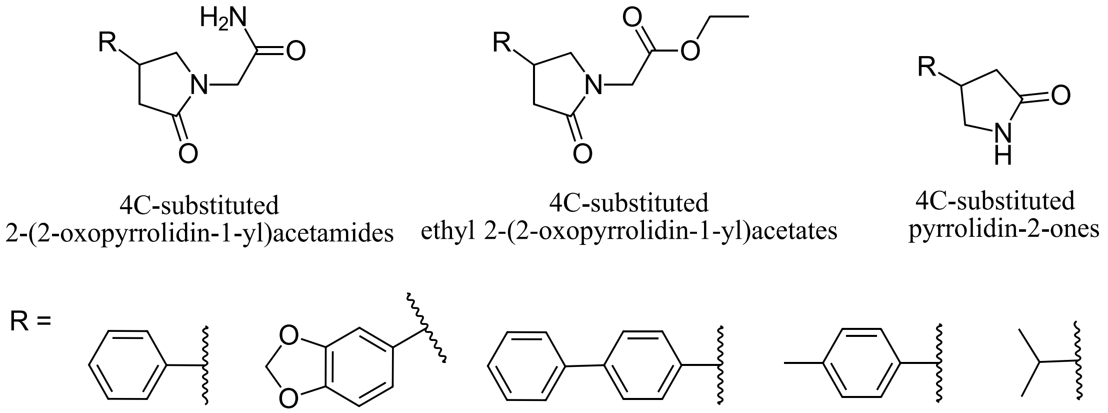

Figure S18. Chemical structure of 4C-substituted pyrrolidin-2-one derivatives (Upmanis, Kažoka, Orlova, & Vorona, 2020)

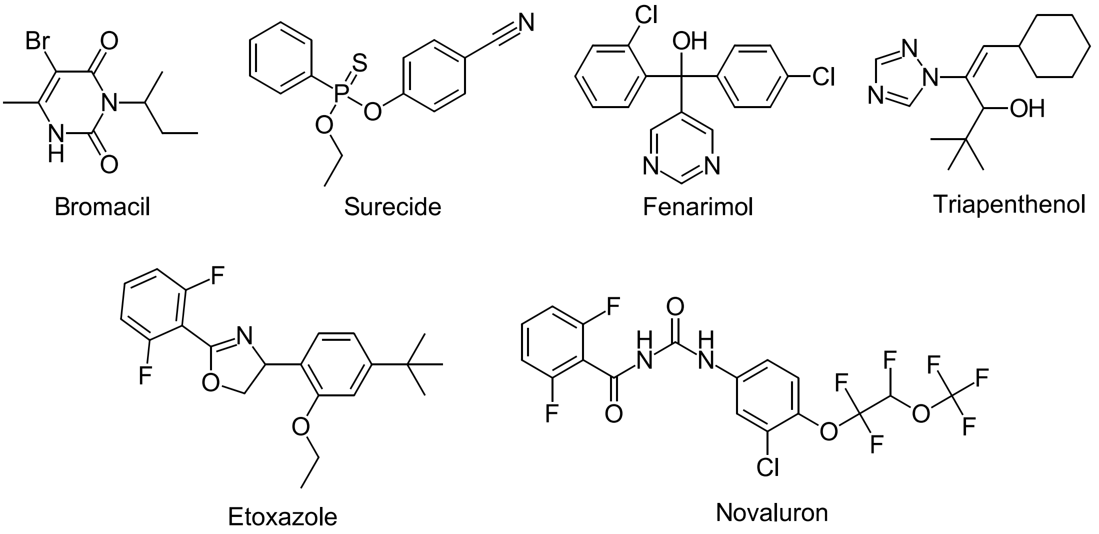

Figure S19. The chemical structures of chiral pesticides (P. Zhao, Li, Chen, Guo, & Zhao, 2019)

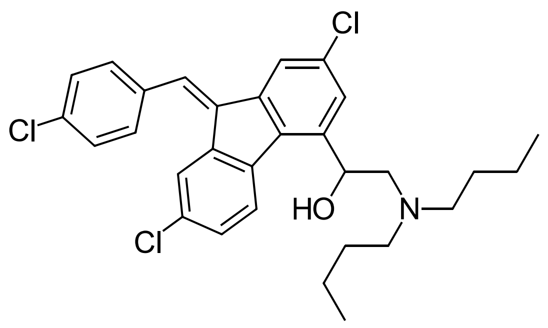

Figure S20. The chemical structure of lumefantrine (Kim et al., 2019)

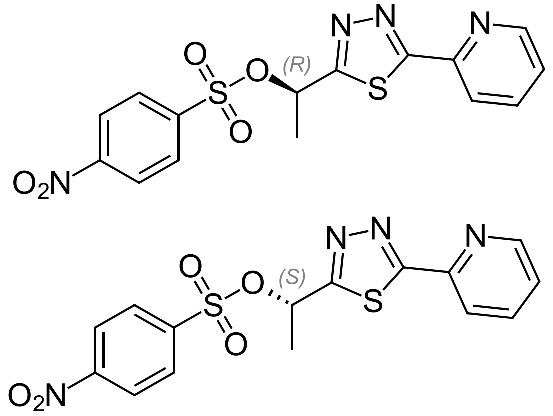

Figure S21. The chemical structure of 5-[1-(4-nitrobenzylsulfonyloxy)-ethyl]-5-(pyridine-2-yl)-[1,3,4]-thiadiazole (Rane et al., 2019)

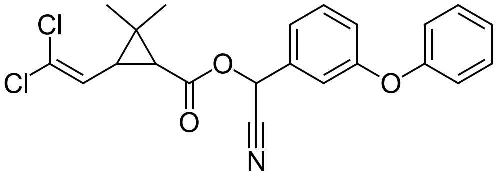

α-cypermethrin

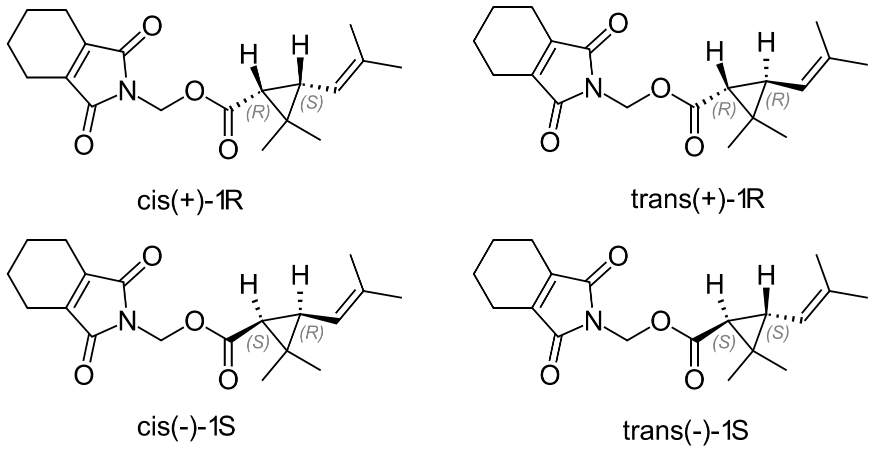

tetramethrin

Figure S22. Structures of α-cypermethrin and tetramethrin

(P. Zhao, Dong, Chen, Guo, & Zhao, 2019)

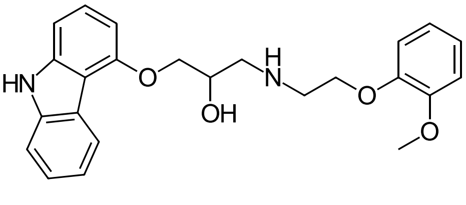

Figure S23. Chemical structure of carvedilol (Panella, Ferretti, Casulli, & Cirilli, 2019)

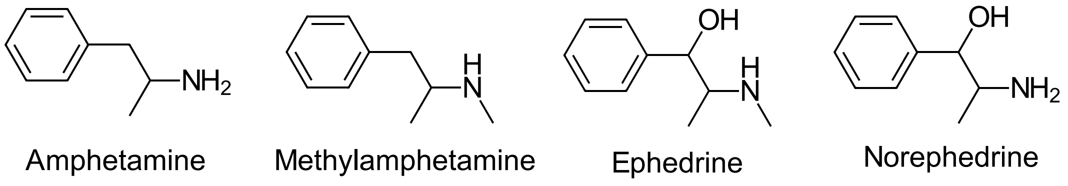

Figure S24. The chemical structures of amphetamine, pseudoephedrine, and related compounds (Karakka Kal et al., 2019)

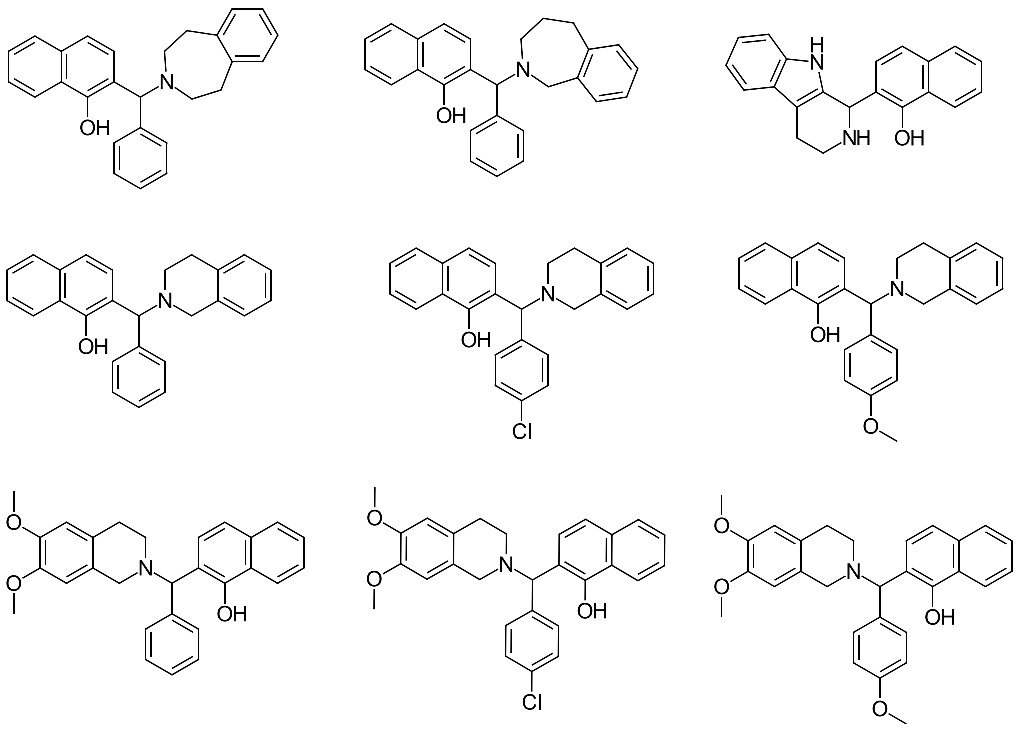

Figure S25. Chemical structures of the chiral amino compounds (Bajtai et al., 2019)

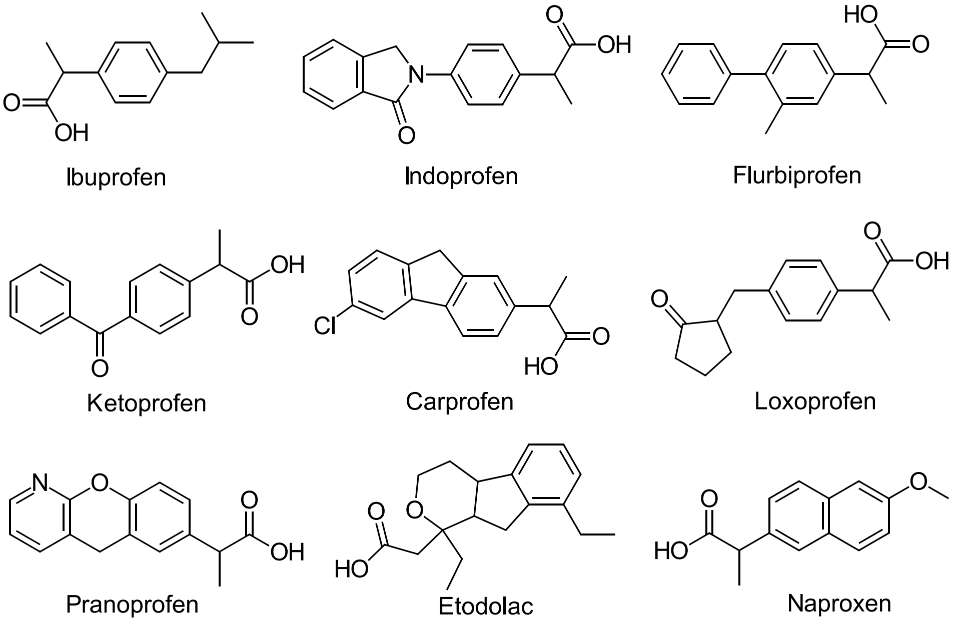

Figure S26. Chemical structures of nine profens studied in fish tissue (M. Li, Liang, Guo, Di, & Jiang, 2020)

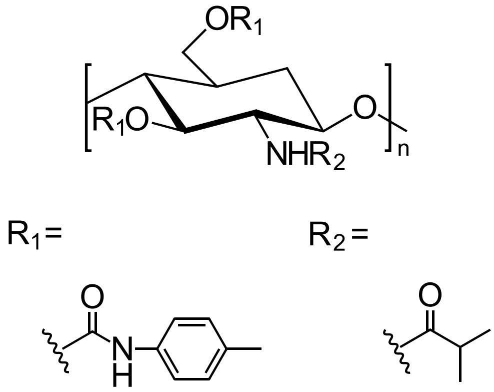

Figure S27. Chemical structure of chitosan 3,6-*bis*-(4-methylphenyl carbamate)-2-(isobutyrylamide)-based CSs (G.-H. Zhang, Xi, Chen, & Bai, 2020)

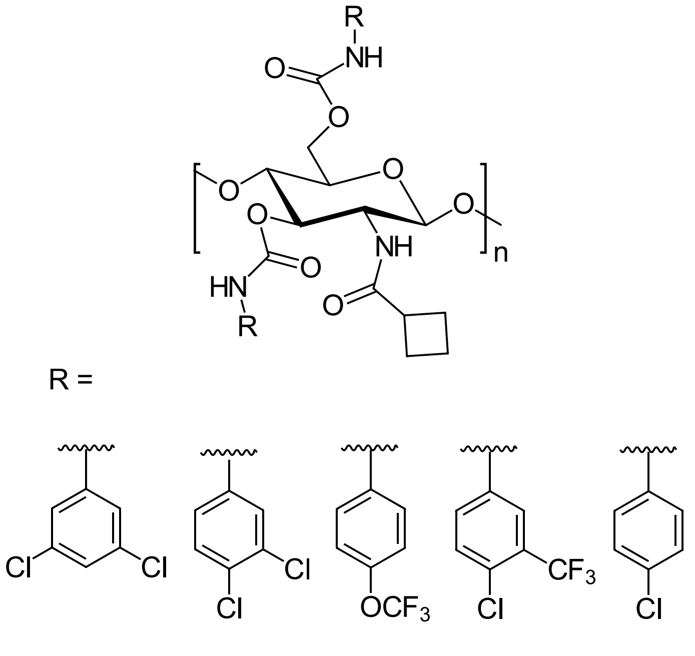

Figure S28. Chemical structure of chitosan 3,6-*bis*(phenylcarbamate)-2-(cyclobutylformamide)-based CSs (J. Zhang, Zhang, Wang, Bai, & Chen, 2019)

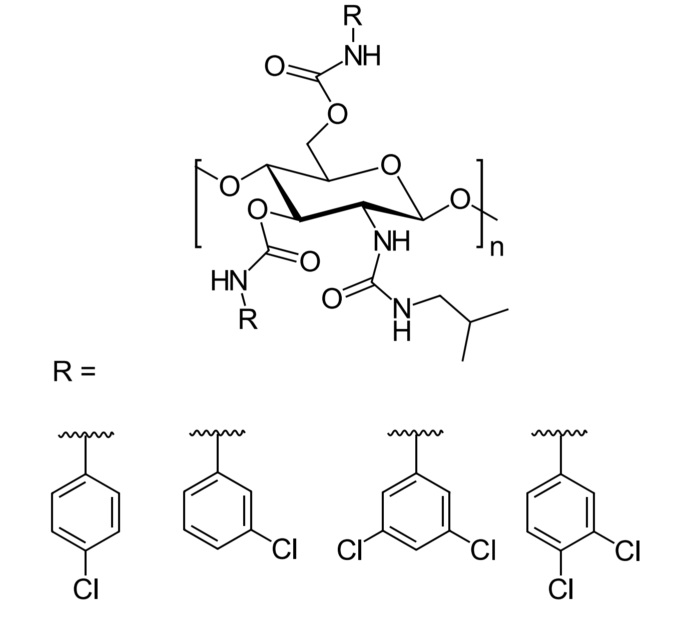

Figure S29. Chemical structure of the chitosan derivatives-based CSs (G.-H. Zhang, Liang, Tang, Chen, & Bai, 2019)

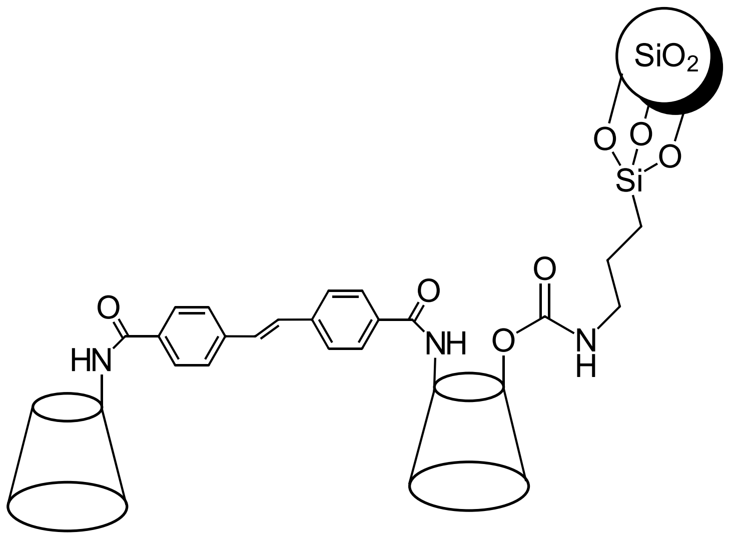

Figure S30. Chemical structure of the stilbene diamido-bridged *bis*-(β-cyclodextrin) bonded silica gel-based CSP (Shuang, Zhang, & Li, 2020)

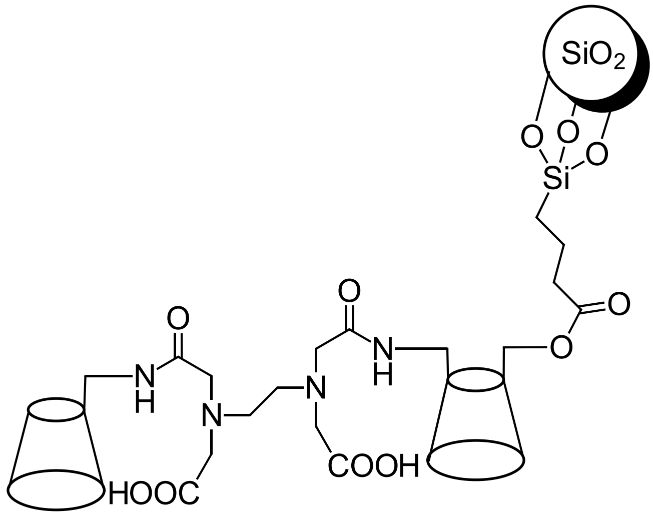

Figure S31. Chemical structure of an ethylenediamine dicarboxyethyl diacetamido-bridged *bis*-(β-cyclodextrin) bound to silica gel as CSP (Shuang, Liao, Zhang, & Li, 2020)

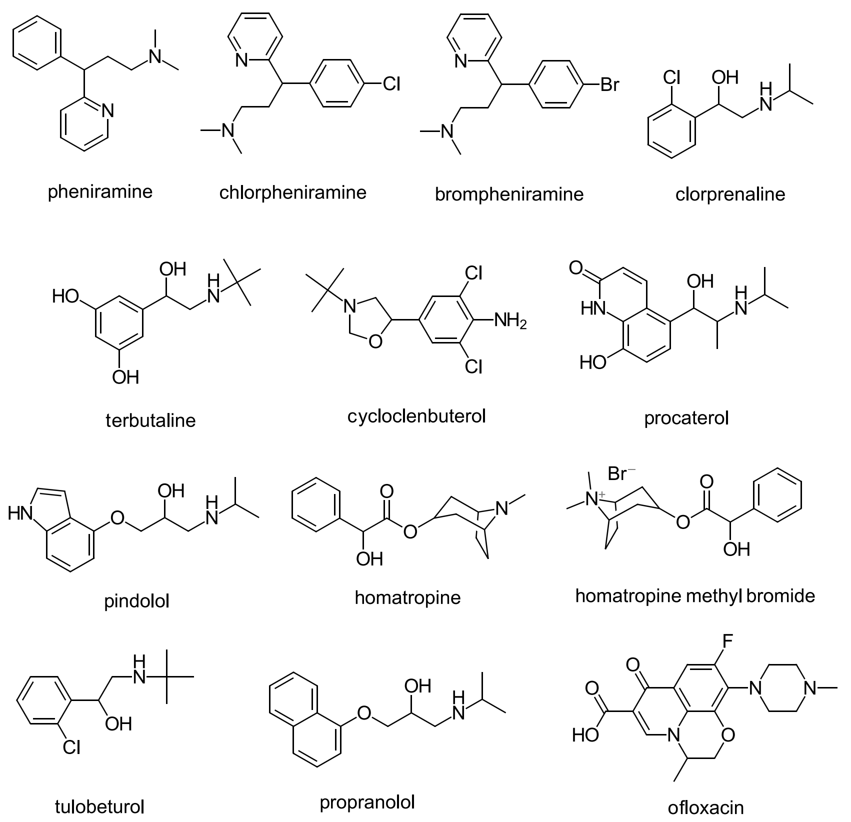

Figure S32. Structures of the chiral analytes used in the study of Zhao *et al.* (Y. Zhao et al., 2020)

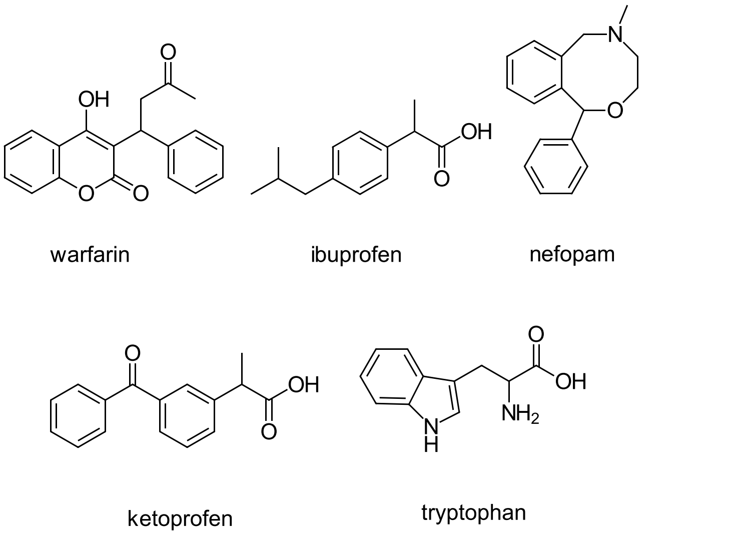

Figure S33. Chemical structure of chiral analytes used in the study of Ke *et al.* (Ke et al., 2020)

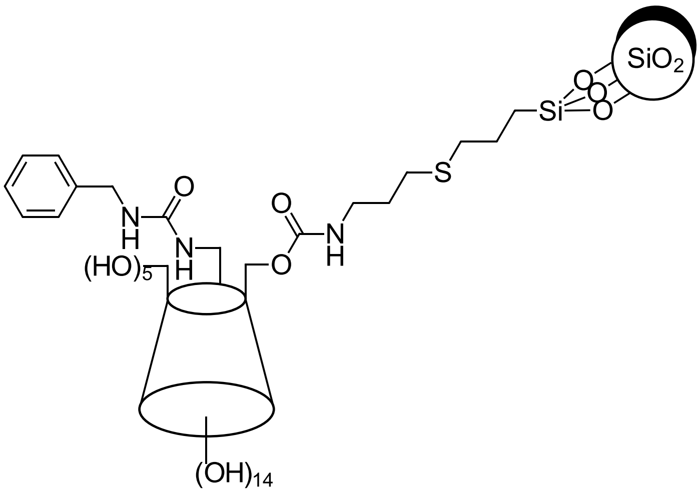

Figure S34. Chemical structure of benzylureido-β-cyclodextrin bound to silica gel as CSP (L.Li, Wang, Jin, Shuang, & Li, 2019)

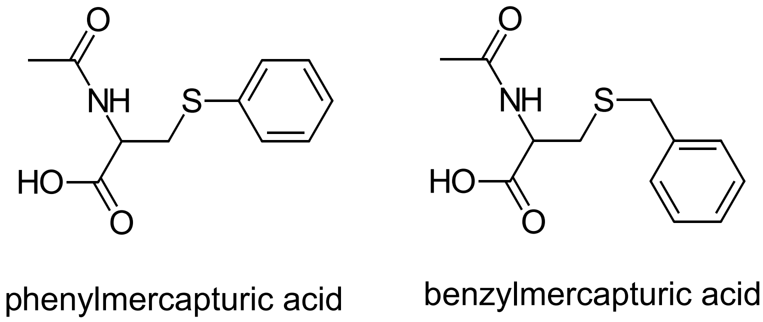

Figure S35. Chemical structure of phenylmercapturic and benzylmercapturic acid (L. Li, Wang, Jin, et al., 2019)

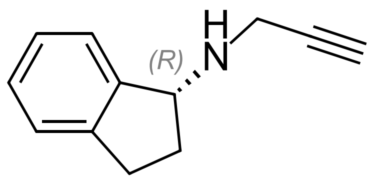

Figure S36. Chemical structure of rasagiline

(Szabó, Ludmerczki, Fiser, Noszál, & Tóth, 2019)

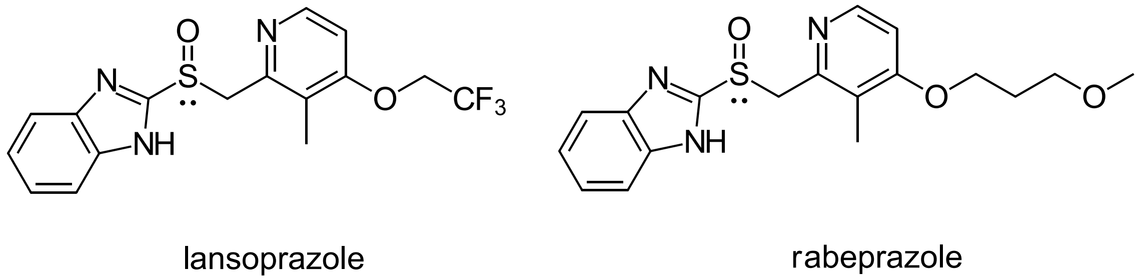

Figure S37. The chemical structure of lansoprazole and rabeprazole

(Papp et al., 2019)

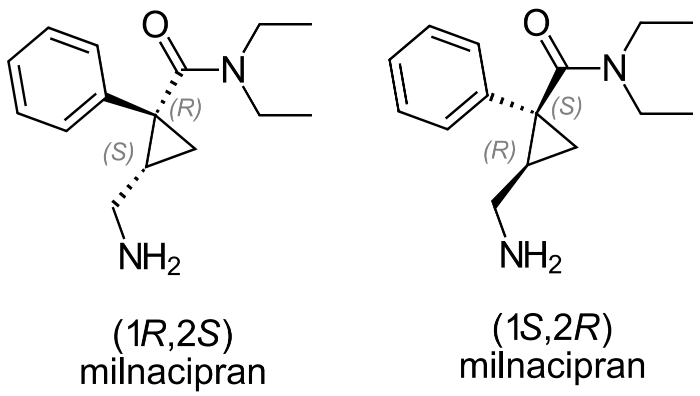

Figure S38. Chemical structure of milnacipran

(Pathak, Coutinho, Mohanraj, Martis, & Jain, 2020)

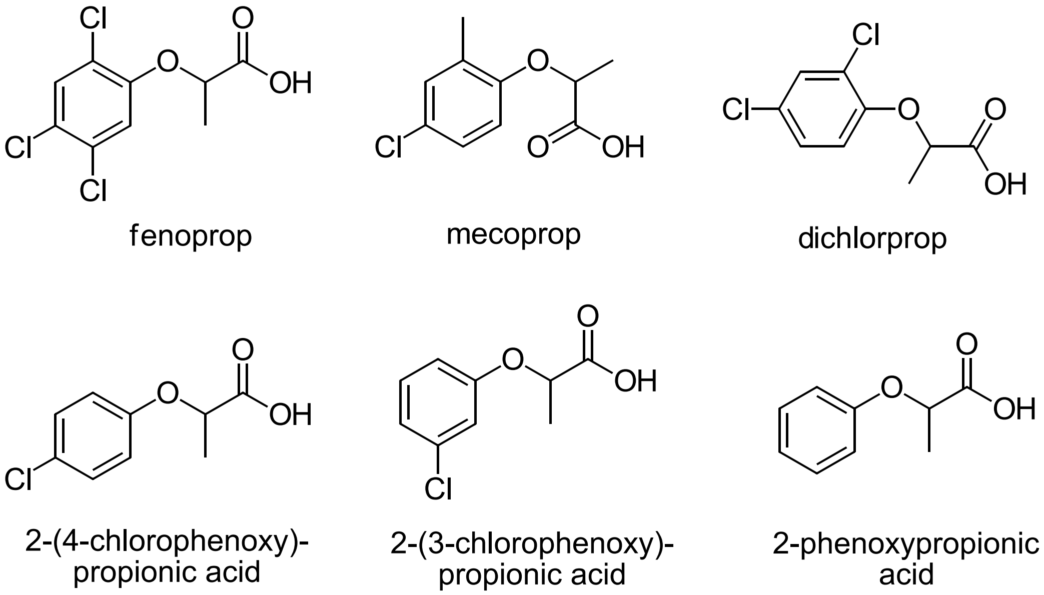

Figure S39. Chemical structure of the chiral analytes used in the study of Casado *et al.* (Casado, Saz, García, & Marina, 2020)

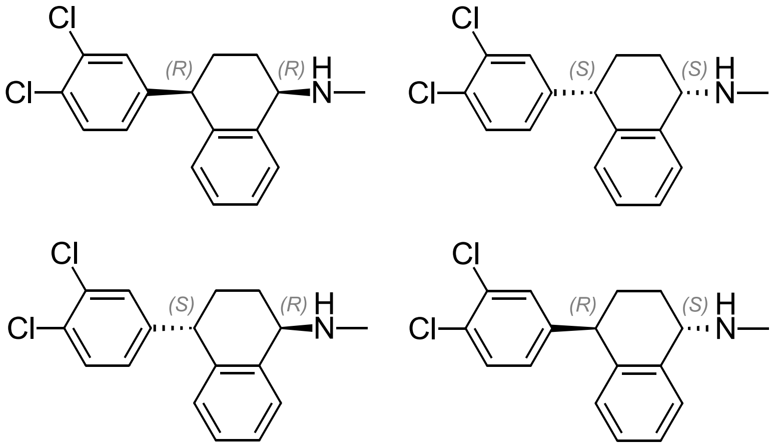

Figure S40. Chemical structures of sertraline isomers (Sun et al., 2019)

References

Amer, H., Nypelö, T., Sulaeva, I., Bacher, M., Henniges, U., Potthast, A., & Rosenau, T. (2016). Synthesis and characterization of periodate-oxidized polysaccharides: dialdehyde xylan (DAX). *Biomacromolecules,* 17(9), 2972-2980.

Bajtai, A., Lajkó, G., Németi, G., Szatmári, I., Fülöp, F., Péter, A., & Ilisz, I. (2019). High-performance liquid chromatographic and subcritical fluid chromatographic separation of α-arylated ß-carboline, N-alkylated tetrahydroisoquinolines and their bioisosteres on polysaccharide-based chiral stationary phases. *Journal of separation science,* 42(17), 2779-2787.

Carradori, S., Secci, D., Guglielmi, P., Pierini, M., & Cirilli, R. (2020). High-performance liquid chromatography enantioseparation of chiral 2-(benzylsulfinyl) benzamide derivatives on cellulose tris (3, 5-dichlorophenylcarbamate) chiral stationary phase. *Journal of Chromatography A,* 1610, 460572.

Casado, N., Saz, J. M., García, M. Á., & Marina, M. L. (2020). Modeling-based optimization of the simultaneous enantiomeric separation of multicomponent mixtures of phenoxy acid herbicides using dual cyclodextrin systems by Capillary Electrophoresis. *Journal of Chromatography A,* 1610, 460552.

Cerra, B., Macchiarulo, A., Carotti, A., Camaioni, E., Varfaj, I., Sardella, R., & Gioiello, A. (2020). Enantioselective HPLC Analysis to Assist the Chemical Exploration of Chiral Imidazolines. *Molecules,* 25(3), 640.

Cheng, L., Cai, J., Fu, Q., & Ke, Y. (2019). Efficient preparative separation of 6-(4-aminophenyl)-5-methyl-4, 5-dihydro-3 (2H)-pyridazinone enantiomers on polysaccharide-based stationary phases in polar organic solvent chromatography and supercritical fluid chromatography. *Journal of separation science,* 42(15), 2482-2490.

Dai, X., Bi, W., Sun, M., Wang, F., Shen, J., & Okamoto, Y. (2019). Chiral recognition ability of amylose derivatives bearing regioselectively different carbamate pendants at 2, 3-and 6-positions. *Carbohydrate polymers,* 218, 30-36.

Dascalu, A.-E., Ghinet, A., Chankvetadze, B., & Lipka, E. (2020). Comparison of dimethylated and methylchlorinated amylose stationary phases, coated and covalently immobilized on silica, for the separation of some chiral compounds in supercritical fluid chromatography. *Journal of Chromatography A,* 461053.

Ferencz, E., Kovács, B., Boda, F., Foroughbakhshfasaei, M., Kelemen, É. K., Tóth, G., & Szabó, Z.-I. (2020). Simultaneous determination of chiral and achiral impurities of ivabradine on a cellulose tris (3-chloro-4-methylphenylcarbamate) chiral column using polar organic mode. *Journal of pharmaceutical and biomedical analysis,* 177, 112851.

Gao, J., Chen, L., Wu, Q., Li, H., Dong, S., Qin, P., Yang, F., & Zhao, L. (2019). Preparation and chromatographic performance of a multifunctional immobilized chiral stationary phase based on dialdehyde microcrystalline cellulose derivatives. *Chirality,* 31(9), 669-681.

Ianni, F., Blasi, F., Giusepponi, D., Coletti, A., Galli, F., Chankvetadze, B., Galarini, R., & Sardella, R. (2020). Liquid chromatography separation of α-and γ-linolenic acid positional isomers with a stationary phase based on

covalently immobilized cellulose tris (3, 5-dichlorophenylcarbamate). *Journal of Chromatography A,* 1609, 460461.

Karakka Kal, A. K., Karatt, T. K., Sayed, R., Philip, M., Meissir, S., & Nalakath, J. (2019). Separation of ephedrine and pseudoephedrine enantiomers using a polysaccharide-based chiral column: A normal phase liquid chromatography–high-resolution mass spectrometry approach. *Chirality,* 31(8), 568-574.

Karakka Kal, A. K., Nalakath, J., Kunhamu Karatt, T., Perwad, Z., Mathew, B., & Subhahar, M. (2020). Development and validation of a chiral LC-MS method for the enantiomeric resolution of (+) and (−)-medetomidine in equine plasma by using polysaccharide-based chiral stationary phases. *Chirality,* 32(3), 314-323.

Ke, J., Zhang, Y., Zhang, X., Liu, Y., Ji, Y., & Chen, J. (2020). Novel chiral composite membrane prepared via the interfacial polymerization of diethylamino-beta-cyclodextrin for the enantioseparation of chiral drugs. *Journal of Membrane Science,* 597, 117635.

Kim, T., Bao, C., Hausmann, M., Siqueira, G., Zimmermann, T., & Kim, W. S. (2019). 3D Printed Disposable Wireless Ion Sensors with Biocompatible Cellulose Composites. *Advanced Electronic Materials,* 5(2), 1800778.

Li, L., Wang, H., Jin, Y., Shuang, Y., & Li, L. (2019). Preparation of a new benzylureido-β-cyclodextrin-based column and its application for the determination of phenylmercapturic acid and benzylmercapturic acid enantiomers in human urine by LC/MS/MS. *Analytical and bioanalytical chemistry,* 411(21), 5465-5479.

Li, L., Wang, H., Shuang, Y., & Li, L. (2019). The preparation of a new 3,5-dichlorophenylcarbamated cellulose-bonded stationary phase and its application for the enantioseparation and determination of chiral fungicides by LC-MS/MS. *Talanta,* 202, 494-506.

Li, M., Jiang, Z., Di, X., & Song, Y. (2020). Enantiomeric separation of six beta-adrenergic blockers on Chiralpak IB column and identification of chiral recognition mechanisms by molecular docking technique. *Biomedical Chromatography*, 34, e4803.

Li, M., Liang, X., Guo, X., Di, X., & Jiang, Z. (2020). Enantiomeric separation and enantioselective determination of some representive non-steroidal anti-inflammatory drug enantiomers in fish tissues by using chiral liquid chromatography coupled with tandem mass spectrometry. *Microchemical Journal,* 153, 104511.

Luo, X., Fang, C., Mi, J., Xu, J., & Lin, H. (2019). Enantiomeric resolution, thermodynamic parameters, and modeling of clausenamidone and neoclausenamidone on polysaccharide-based chiral stationary phases. *Chirality,* 31(6), 423-433.

Panella, C., Ferretti, R., Casulli, A., & Cirilli, R. (2019). Temperature and eluent composition effects on enantiomer separation of carvedilol by high-performance liquid chromatography on immobilized amylose-based chiral stationary phases. *Journal of Pharmaceutical Analysis,* 9(5), 324-331.

Papp, L. A., Hancu, G., Gyéresi, Á., Kelemen, H., Szabó, Z. I., Noszál, B., Dubský, P., & Tóth, G. (2019). Chiral separation of lansoprazole and rabeprazole by capillary electrophoresis using dual cyclodextrin systems. *Electrophoresis,* 40(21), 2799-2805.

Pathak, P., Coutinho, E. C., Mohanraj, K., Martis, E., & Jain, V. (2020). Chromatographic and Computational Studies on the Chiral Recognition of Sulfated β-Cyclodextrin on Enantiomeric Separation of Milnacipran. *ChemRxiv.* Preprint. https://doi.org/10.26434/chemrxiv.11726196.v2.

Rane, V. P., Ahirrao, V. K., Patil, K. R., Jadhav, R. A., Ingle, R. G., More, K. B., & Yeole, R. D. (2019). Enantiomeric Separation and Thermodynamic investigation of (R)-5-[1-(4-Nitrobenzylsulfonyloxy)-ethyl]-5-(pyridine-2-yl)-[1, 3, 4]-thiadiazole, a Key Intermediate of Nafithromycin. *Analytical Chemistry Letters,* 9(5), 625-633.

Shuang, Y., Liao, Y., Zhang, T., & Li, L. (2020). Preparation and evaluation of an ethylenediamine dicarboxyethyl diamido-bridged bis (β-cyclodextrin)-bonded chiral stationary phase for high performance liquid chromatography. *Journal of Chromatography A*, 460937.

Shuang, Y., Zhang, T., & Li, L. (2020). Preparation of a stilbene diamido-bridged bis (β-cyclodextrin)-bonded chiral stationary phase for enantioseparations of drugs and pesticides by high performance liquid chromatography. *Journal of Chromatography A,* 1614, 460702.

Sun, W., Wang, C., Jin, Y., Wang, X., Zhao, S., Luo, M., . . . Tong, S. (2019). Stereoselective separation of (1S, 4S)-sertraline from medicinal reaction mixtures by countercurrent chromatography with hydroxypropyl-β-cyclodextrin as stereoselective selector. *Journal of separation science,* 42(16), 2734-2742.

Szabó, Z. I., Ludmerczki, R., Fiser, B., Noszál, B., & Tóth, G. (2019). Chiral separation of rasagiline using sulfobutylether-β-cyclodextrin: capillary electrophoresis, NMR and molecular modeling study. *Electrophoresis,* 40(15), 1897-1903.

Tantawy, M. A., Yehia, A. M., & Aboul-Enein, H. Y. (2019). Simultaneous determination of guaifenesin enantiomers and ambroxol HCl using 50-mm chiral column for a negligible environmental impact. *Chirality,* 31(10), 835-844.

Upmanis, T., Kažoka, H., Orlova, N., & Vorona, M. (2020). Separation of 4C-Substituted Pyrrolidin-2-One Derivatives on Polysaccharide-Based Coated Chiral Stationary Phases. *Chromatographia,* 83(3), 331-340.

Wang, H., Wang, Q., Wu, Y., Cheng, L., Zhu, L., Zhu, J., & Ke, Y. (2019). HPLC and SFC enantioseparation of (±)-Corey lactone diol: Impact of the amylose tris-(3, 5-dimethylphenylcarbamate) coating amount on chiral preparation. *Chirality,* 31(10), 855-864.

Yin, C., Chen, W., Zhang, J., Zhang, M., & Zhang, J. (2019). A facile and efficient method to fabricate high-resolution immobilized cellulose-based chiral stationary phases via thiol-ene click chemistry. *Separation and Purification Technology,* 210, 175-181.

Yu, X., Wang, Y., Yang, Q., Zhang, Z., Ren, Q., Bao, Z., & Yang, Y. (2020). De novo synthesis of microspheical cellulose 3, 5-dichlorophenylcarbamates: An organic-inorganic hybrid chiral stationary phase for enantiospearation. *Separation and Purification Technology,* 238, 116480.

Zhang, G.-H., Liang, S., Tang, S., Chen, W., & Bai, Z.-W. (2019). Performance evaluation of enantioseparation materials based on chitosan isobutylurea derivatives. *Analytical methods,* 11(12), 1604-1612.

Zhang, G.-H., Xi, J.-B., Chen, W., & Bai, Z.-W. (2020). Comparison in enantioseparation performance of chiral stationary phases prepared from chitosans of different sources and molecular weights. *Journal of Chromatography A*, 461029.

Zhang, J., Sun, J., Liu, Y., Yu, J., & Guo, X. (2019). Immobilized Cellulose-Based Chiralpak IC Chiral Stationary Phase for Enantioseparation of Eight Imidazole Antifungal Drugs in Normal-Phase, Polar Organic Phase and Reversed-Phase Conditions Using High-Performance Liquid Chromatography. *Chromatographia,* 82(3), 649-660.

Zhang, J., Zhang, G.-H., Wang, X.-C., Bai, Z.-W. & Chen, W (2019). Synthesis and evaluation of novel chiral stationary phases based on N-cyclobutylcarbonyl chitosan derivatives. *Microchemical Journal*, 147, 224-231.

Zhao, P., Dong, X., Chen, X., Guo, X., & Zhao, L. (2019). Stereoselective Analysis of Chiral Pyrethroid Insecticides Tetramethrin and α-Cypermethrin in Fruits, Vegetables, and Cereals. *Journal of agricultural and food chemistry*, 67(33), 9362-9370.

Zhao, P., Li, S., Chen, X., Guo, X., & Zhao, L. (2019). Simultaneous enantiomeric analysis of six chiral pesticides in functional foods using magnetic solid-phase extraction based on carbon nanospheres as adsorbent and chiral liquid chromatography coupled with tandem mass spectrometry. *Journal of pharmaceutical and biomedical analysis*, 175, 112784.

Zhao, Y., Wang, J., Liu, Y., Jiang, Z., Song, Y., & Guo, X. (2020). Enantioseparation using carboxymethyl-6-(4-methoxybenzylamino)-β-cyclodextrin as a chiral selector by capillary electrophoresis and molecular modeling study of the recognition mechanism. *New Journal of Chemistry*, 44, 958-972.
